# Pre-conceptional paternal diet impacts on offspring testosterone homoeostasis via epigenetic modulation of *cyp19a1/aromatase* activity

**DOI:** 10.1038/s44324-024-00011-8

**Published:** 2024-06-17

**Authors:** Arianna Pastore, Nadia Badolati, Francesco Manfrevola, Serena Sagliocchi, Valentina Laurenzi, Giorgia Musto, Veronica Porreca, Melania Murolo, Teresa Chioccarelli, Roberto Ciampaglia, Valentina Vellecco, Mariarosaria Bucci, Monica Dentice, Gilda Cobellis, Mariano Stornaiuolo

**Affiliations:** 1 Department of Pharmacy, University of Naples “Federico II”, Via Montesano 49, 80131 Naples, Italy; 2 Telethon Institute of Genetics and Medicine (TIGEM), Via Campi Flegrei, 34, 80078 Pozzuoli, Italy; 3 Department of Experimental Medicine, University della Campania “Luigi Vanvitelli”, Via Santa Maria di Costantinopoli 16, 80138 Naples, Italy; 4 Department of Clinical Medicine and Surgery, Via Sergio Pansini 5, University of Naples “Federico II”, 80131 Naples, Italy

**Keywords:** Metabolic disorders, Cell biology

## Abstract

Paternal eating habits, before and at conception, have a strong impact on offspring future metabolism. By sending specific epigenetic signals through spermatozoa, paternal nutrition influences developing embryos and increases offspring risk of developing dysmetabolism and cardiovascular diseases. Among the intergenerational consequences, paternal epigenetic messages affect embryo DNA methylation altering programmed gene expression. The identification of offspring genetic loci that are epigenetically altered by paternal stimuli is of pivotal interest for timely post-natal treatment of offspring metabolic defects. We here use a murine model to show that, *cyp19a1/aromatase*, a gene coding for the cytochrome converting testosterone into 17-β estradiol (both potent hormonal mediators of embryo development and metabolism), is an epigenetic transducer of paternal intergenerational inheritance. By affecting *cyp19a1* methylation status and alternative splicing, paternal diet coordinates androgens’ metabolism in the progeny affecting it in a sexually dimorphic way and promoting hypoandrogenism, growth retardation and diabetes in male pups.

## Introduction

Our predisposition to diseases is part of our phenotype and, as such, is not the mere result of the unique asset of genetic polymorphisms occurring at coding regions and regulatory segments of our genome. Epigenetic factors (genomic differences other than variations of the nucleotide sequences) influence enormously the phenotypic aspect of an individual^1,2^. The epigenetic fingerprint of the genome includes: (i) covalent modification of nitrogen bases (DNA methylation and hydroxy-methylation) or of histones (e.g. methylation and acetylation)^3–5^, (ii) chromosome three-dimensional organisation (supramolecular assembly promoted by chromatin condensation) and (iii) expression of small non-coding RNAs^6–8^.

Two characteristics of epigenetics are particularly attractive for epidemiologists. First, like the genome, the epigenome is inheritable. The epigenetic state of male and female germ cells are important vehicles of parental information for the zygote. These will influence the zygote during embryo development and, after birth, its propensity to develop diseases^9^. A second interesting aspect of the epigenome is its great susceptibility to environmental stimuli. Unlike the genome, which requires powerful chemical reactants to be modified (X-ray, UV radiation and alkylating agents), the epigenome is easily altered by the environment. Susceptibility is such that, here, the term “environment” must be used in its broadest meaning. The epigenome is, indeed, influenced by metabolic factors (intracellular flux or availability of metabolites, drugs, eating habits and metabolic fitness) and environmental parameters in the strict sense (presence of pollutants, social relationships, mental and relational stress)^10^.

Probably to avoid this epigenetic susceptibility and the consequent massive effect that the environment would have on the epigenome of future generations, the processes of oogenesis and spermatogenesis as well as of embryo development are interspersed by phases of epigenetic signals’ removal and reshuffling^11^. Before fertilisation, demethylation events populate primordial germ cells differentiation into spermatogonia and primary oocytes. Post-fertilisation, during a phase of zygote active demethylation, demethylase removes methyl groups from cytosines of paternal chromosomes. After the completion of the first cell cycle of the zygote, DNA methylation continues to decline due to DNA methylase inactivity^12^. Nonetheless, some genomic regions escape demethylation, preserving or, more often, reacquiring the methylation status of the parents, ultimately imprinting the offspring genome and influencing their adult lives^13^.

Paternal intergenerational inheritance of dysmetabolism has found evidence in almost all eukaryotic models^5,14,15^. High-calorie diets administered to the parental generation F0 is one of the most described environmental stimuli capable of promoting epigenetic alteration in the progeny. Either administered to sires or to mothers, this stimulus affects the epigenome and phenotype of the F1 generation, which often presents, independently from their calorie intake, higher propension to dysmetabolism. Father fed high fat diet (HFD) predicts progeny presenting glucose intolerance and insulin resistance, both in mice and in rats^16^. In humans, epigenetic inheritance has been mostly studied in generations born during and immediately after periods of famine or food excess^17^. In the Överkalix cohort, paternal grandfathers access to food in their slow growth period correlates with their grandchildren’s overall mortality risk, cardiovascular disease, BMI, waist circumference and fat mass^18^. Recently, evidence of paternal intergenerational transmission has been confirmed in other cohorts^19^.

Despite the reproducibility of paternal intergenerational transmission, its outcome (penetrance, offspring phenotype, as well as allocation of the epigenetic modification in the F1 epigenomes) can vary. Fathers fed HFD has been shown to promote weight gain or growth retardation^20^, increased or reduced lean mass, diabetes or increased cardiovascular risk depending on the diet, the experimental conditions^21–23^ or the murine strain used^4,7^. Moreover, intergenerational epigenetic studies must deal with several confounding factors. As example, HFD exerts a negative effect on fathers. Sires fed HFD acquire an obese phenotype (mostly characterised by hyperinsulinemia and glucose intolerance). By promoting the expansion of adipose tissue, obesity induces testosterone reduction and impaired spermatogenesis^24^ along with a reduction in mating efficiency and fertility^25^. Disinterest in mating is one of the most common confounding factors in experimental paradigms addressing intergenerational inheritance of paternal stimuli. Indeed, male disinterest represents a stress for the females and thus, constitutes itself an environmental stimulus, recorded by the oocyte and transmitted to the future generation^26^.

Many aspects of paternal epigenetic inheritance remain still obscure. Among the others, it remains to be fully identified which gene regions in the progeny may be a depository of information coming from the parents. These genomic loci might either be: i) imprinted control regions (ICR), i.e. young transposon families, usually located far from gene loci, or ii) differentially methylated regions (DMR) close to the gene regions^27^. Both have been shown to elude the demethylating waves occurring during fertilisation or to acquire a specific epigenetic state during embryo development. The identification of these regions would help reveal the metabolic mechanism underpinning intergenerational inheritance, and, more importantly, identify cohorts of children more susceptible to dysmetabolism. Although not all supported by the same scientific evidence, several genetic loci have been shown to be epigenetically affected by environmental stimuli recorded by parents, including (among the others) hormone receptor (corticosteroid, leptin and oxytocin), growth factors of the Insulin growth factor family, IGF-I and IGF-II, and their cognate receptors^28^.

Here, we show in a murine model, that an environmental stimulus corresponding to HFD administered for eight weeks to sires, results in the epigenetic modulation of offspring *cyp19a1*, a gene coding for the enzyme Aromatase and responsible for converting testosterone into 17-β estradiol. We show that this epigenetic alteration promotes the activity of the enzyme and results in the decrease of testosterone levels in offspring testis, ultimately impairing the growth of male pups and predisposing them to hyperinsulinemia and glucose intolerance in adulthood. Interestingly, the effect is sexually dimorphic, with females positively influenced by the epigenetic modulation and presenting normal weight and improved glucose tolerance.

## Methods

### Animals

All animal procedures were approved by Institutional Animal Care and Use Committee (IACUC) (protocol n° 846/2020-PR). All animal experiments complies with the ARRIVE guidelines (see Supplementary Information) and were carried out in accordance with the U.K. Animals (Scientific Procedures) Act, 1986 and associated guidelines, EU Directive 2010/63/EU for animal experiments. All animals, C57BL/6 J Ola mouse (Envigo, Europe), were housed 5 per cage under controlled conditions and 12:12-h light-dark cycle. After two weeks of acclimatation, 5-weeks-old F0 male were split into two groups of equal average body weight. Control mice (F0-CON group, *n* = 30) were fed a control diet (Teklad Rodent Diet, # TD-8604: 3; Envigo, Europe). The high fat diet (F0-HFD group, *n* = 30) group was fed HFD (Teklad Rodent Diet, # TD-06414: 5.1 kcal/g, 60.3% fat, 18.3% protein, and 21.4% carbohydrate; Envigo, Europe). After 8 weeks of diet, F0 males from each group were mated with 13-weeks-old C57BL/6 J Ola females (*n* = 120) consuming control diet. Females were nulliparous, non-diabetic and did not differ in body weight, fasting blood glucose, or plasma insulin concentration, as measured 1 week before mating (Supplementary Table 1). During mating, one male and two female were housed together with *ad libitum* access to food. Mating was verified by vaginal plug. F0 males were culled after overnight fasting, one week after mating.

### Mating and offspring

From the 120 mated pairs, 90 resulted in a successful pregnancy (43 and 47 for F0-CON and F0-HFD respectively). Females were fed CON diet throughout pregnancy and lactation. To control for postnatal nutrition, litter size was immediately reduced to 5 pups where necessary. The selection of pups to remove was done randomly. After weaning, one female and one male pup from each litter were randomly selected and allocated into two new groups: F1 generation from F0-HFD was named F1(F0-HFD) (*n* = 43 per sex, each coming from a different litter) while the generation from F0-CON was named F1(F0-CON) (*n* = 43 per sex, each coming from a different litter). Independently from their fathers, male and female offspring were fed control diet (14% kcal fat) from weaning up to week 13, when all underwent metabolic testing, biochemical analyses, and post-mortem necropsy. Average weight of the animals, before and after allocation or selection is reported in the manuscript as Supplementary Table 1 and Supplementary Fig. 4. F1 offspring were culled at 16 weeks of age. Tissues and blood of culled offspring were snap-frozen in liquid nitrogen and then stored at −80 °C for further analysis. Blood and tissues were collected, between 9 AM and 1 PM, with groups randomised across this time to account for differences in fasted state and diurnal hormone secretion.

### Oral glucose tolerance test

60 F0 sires (30 per groups) and 172 F1 offspring (43 per groups, per sex) underwent oral glucose tolerance test (OGTT) 2 weeks before cull. OGTT was performed following a 6 h fasting. Two grams of glucose/kg body weight (20% wt/vol glucose) was administered by gavage, and blood glucose concentration was measured at 0, 15, 30, 60, 90, and 120 min using a Contour Next Glucose metre (Bayer, Basel, Switzerland). Insulin was measured by ELISA (Insulin Mouse ELISA Kit, # ab277390, Invitrogen, Thermo Fisher Scientific, USA). Plasma total cholesterol, high-density lipoprotein cholesterol (HDL), low-density lipoprotein cholesterol (LDL), triglyceride, aspartate aminotransferase (AST) and alanine aminotransferase (ALT) were determined using commercially available kits from Diacron International (Grosseto, Italy). Analyses were performed on a Diacron International Free Carpe Diem analyser.

### Gross necropsy and sperm analysis

Gross necropsy and organ weight were performed according to standard procedures. For each animal, testes were rapidly removed and properly stored at −80 °C or fixed in Bouin’s solution, for molecular and histological analyses, respectively. Epididymis was dissected in caput, corpus and cauda regions and immediately processed for sperm collection. In brief, the caput, corpus and cauda epididymis were separately immersed in 3 ml PBS (pH 7.6) and loosely cut to drain sperm from the ducts. Then, sperm samples were filtered throughout cheesecloth and used for functional and biochemical investigations. Specifically, sperm cells collected from caput (caput sperm), corpus (corpus sperm) and cauda (cauda sperm) epididymis were used to analyse total ROS whereas cauda sperm were used to assay sperm quality parameters (count, motility and viability), mitochondrial ROS and reduced Glutathione (GSH), as reported below.

### Histology analysis

Testes were fixed in Bouin’s solution overnight, dehydrated in ethanol, cleared in xylene, and embedded in paraffin using standard procedures. Tissue sections (7 µm thick) were deparaffined and stained with hematoxylin and eosin (H&E) reagents. Histological analyses were conducted under a light microscope (Leica CTR500, Leica Microsystems Inc., Milan, Italy) and images were captured using a high-resolution digital camera (Leica DC300F) at a magnification of 20X. A minimum of 6 random sections was analysed to evaluate seminiferous epithelium morphology.

### Cauda sperm quality parameters

Sperm number as well as count of motile and viable sperm were evaluated under a light microscope at a magnification of 10X using a hemocytometer (Burker Chamber). The sperm number was expressed as 10^6^ cells/ml. Sperm viability, assessed through the viable dye trypan blue reagent (trypan blue, 0.4% solution, 17-942E, Lonza Bioscience, Walkersville, Maryland, USA), was expressed as percentage of viable/total sperm, while motility was expressed as percentage of motile/total sperm. A minimum of 100 sperm cells were evaluated and counted for each analysis.

### Measurement of oxidative stress in spermatozoa

To measure oxidative stress in spermatozoa, total ROS, mitochondrial ROS and total reduced Glutathione (GSH) were quantitated with Dichlorofluoresceine diacetate (5 μM working solution, CM-H2DCFDA, # C6827, Invitrogen, Waltham, Massachusetts, USA), Mitotracker CMX-ROS (10 μM working solution, MitoTracker Red CMXRos, # M7512, Invitrogen), TAMRA-ioadoacetamide (10 μM, TMR-IAA, # T6006, Invitrogen), respectively. The probes were dissolved in DMSO and stored at −20 °C. A 4× concentrated dilutions of the stocks were freshly prepared the day of the experiments. Spermatozoa were centrifuged at 800 g at RT in a tabletop centrifuge to be then resuspended in 1 mL of PBS. In total, 75 μL of the sperm dilution in PBS was supplemented with 25 μL of 4× probe solution and incubated at 37 °C for 30 min, to be then fixed in PFA 3.7% and analysed under a IRiS™ Digital Cell Imaging System, Fluorescence microscope, (Logos Biosystems, South Korea).

### Testosterone and 17-β estradiol quantitation in testis

Testes were homogenised in 70% MetOH, left on ice for 1 h and then placed at 4 °C for 16 h. Steroids were extracted in ice cold Diethyl ether for 2 min by vortexing. To isolate the organic fraction, samples were frozen in a dry ice/acetone bath. Upon freezing of the MetOH phase, the liquid ether phase was transferred in a new clean glass tube. Organic fractions were dried in a speed vac at 37 °C and resuspended in 1 ml of PBS supplemented with 0.1% gelatin. Testosterone and 17-β estradiol were quantitated by ELISA using the Testosterone ELISA KIT (# RE52631, Tecan, Switzerland) and the 17-β estradiol ELISA KIT (# 30121045, Tecan, Switzerland), respectively using standard curves as suggested by the manufacturer.

### Pancreas histology and immunostaining

Pancreases were dissected and fixed in formalin for 48 hours. Following fixation, samples were washed twice in 0.9% NaCl for 1.5 h and slowly dehydrated by incubation in 50% Ethanol (twice, 1.5 h) and then 70% Ethanol (overnight at 4 °C). The day after, samples were sequentially incubated for 1 h in 85%, 95%, 100% Ethanol (overnight at 4 °C), then in Xylene (twice, 1 h each) and in fresh paraffin (four times, 30 min each before embedding). Tissues were then embedded in tissue mould in fresh paraffin to be then cut it into 10–15 μm sections using a microtome and mounted on microscope slides. For histological and morphological analyses of pancreatic islets, slides were deparaffinized and stained with H&E reagents. Islets’ size and distribution was quantified by *ImageJ* software (version 1.52a, U.S. National Institutes of Health). Up to 12 fields were captured from the same location within each pancreas. The images were acquired by Leica DMi8 microscope (Leica Microsystems, Wetzlar, Germany). For immunohistochemical analyses of pancreatic islets (*n* = 100 islets count per mice), slides were deparaffinized and boiled in citric acid buffer into a microwave oven for antigen retrieval. Sections were then blocked with 3% normal goat serum and 0.3% Triton X-100 in PBS. They were then sequentially incubated with biotinylated primary antibody [(anti-Insulin biotinylated monoclonal Antibody, 1:500; #13-9769-80, eBioscience™, Thermo Fisher Scientific, Waltham, Massachusetts, USA) or (anti-glucagon biotinylated monoclonal Antibody, 1:500; #13-9743-82, eBioscience™, Thermo Fisher Scientific)] overnight at 4 °C followed by HRP-Conjugated Streptavidin (1:1000, # N100, Invitrogen, Thermo Fisher Scientific) for 3 h at room temperature. Immunostaining was finally developed with 3,3’-diaminobenzidine (DAB) (# 34002, Invitrogen, Thermo Fisher Scientific). The images were acquired by Leica DMi8 microscope, (Leica Microsystems, Wetzlar, Germany).

### Western Blot analysis

Livers from F0-CON and F0-HFD mice were homogenised in RIPA buffer. Denatured proteins (40 μg) were separated on 10% SDS polyacrylamide gels and transferred to PVDF membranes and detected by immunoblotting. Membranes were blocked in PBS containing 0.1% vol/vol Tween‐20 and 5% w/vol non‐fat dry milk (# A0830 Applichem GmbH, Darmstadt, Germany) for 45 min, followed by overnight incubation at 4 °C with the following primary antibodies: rabbit p-AKT antibody (Ser 473), 1:1000 (# 4060, Cell Signalling, Beverly, MA, USA); rabbit AKT antibody, 1:1000 (# 9272, Cell Signalling, Beverly, MA, US); rabbit p-AMPK antibody (Thr 172), 1:1000 (# 50081, Cell Signalling, Beverly, MA, US); mouse AMPK 1:1000; (# 2793; Cell Signalling, Beverly, MA, USA). Membranes were extensively washed in PBS containing 0.1% vol/vol Tween‐20 before incubation with anti-mouse (# 115-035-003) and/or anti-rabbit (# 111-035-144) IgG secondary antibodies 1:3000 (Jackson ImmunoResearch, Cambridge, UK) for 90 min at RT. Following incubation, membranes were washed and developed using Chemidoc (Biorad, Hercules, California, US). The target protein band intensity was normalised relative to the intensity of the housekeeping protein mouse monoclonal (β-actin 1:3000; # A 4700, Merck, Darmstadt, Germania). Quantification of results was made using ImageJ Software (version 1.52a, U.S. National Institutes of Health).

### gDNA extraction, methylation analysis by methylation sensitive qPCR

Genomic DNA (gDNA) was extracted from (approximately 25–30 mg) mouse pancreas and testis using the DNeasy Blood and Tissue kit (# 69506, Qiagen, Hilden, Germany) following the manufacturer’s recommended protocol. The concentration of extracted gDNA was determined using a NANODROP 2000 (Thermo Fisher Scientific, Waltham, Massachusetts, USA) spectrophotometer. Additionally, the quality of the gDNA was assessed by subjecting it to electrophoresis on a 1% agarose gel. For gDNA digestion, 80 ng of each DNA sample was supplemented with 1 µL of HpaII (# ER0511, Thermo Fisher) or MspI (# ER0541, Thermo Fisher) in 1× EpiJET Buffer (# K1441, Thermo Fisher) and in a final reaction volume of 20 µL. The digestion was carried out overnight at 37 °C, and the digested products were checked through electrophoresis on a 0.8% agarose gel. 2 µL of each digested DNA was used in PCR reactions, which included 10 µL of QuantiNova Syber Mix (# 208054, Qiagen), 0.6 µL of forward and reverse primers, and 0.3 µL of ROX dye, in a final reaction volume of 20 µL. PCR amplification was performed using the Step One Plus PCR system (Applied Biosystems,) with the following cycling conditions: 95 °C for 3 min followed by 40 cycles of [30 s of denaturation at 95 °C, 30 s of annealing at 52 °C; 30 s of amplification at 60 °C]. The analysis of the methylation state of each target sequence was conducted by comparison amplification Ct of digested samples with undigested control sample following the comparative 2^−∆Ct^ method.

### Sequencing of bisulfite converted gDNAs

Bisulfite conversion of 2 μg of extracted gDNA was performed using the EpiTect Bisulfite Kit (# 59104, Qiagen) following the manufacturing protocol. The conditions for the bisulfite reaction were as follows: Initial step [denaturation: 5 min at 95 °C; incubation: 25 min at 60 °C]; second step [denaturation: 5 min at 95 °C, incubation: 85 min at 60 °C], third step [denaturation: 5 min at 95 °C, incubation: 175 min at 60 °C]. The bisulfite-converted gDNA was subjected to PCR amplification using the Real-Time PCR System StepOnePlus (Applied Biosystems, Thermo Fisher Scientific, Waltham, Massachusetts, USA) using the following amplification steps: 95 °C for 3 min followed by 40 cycles of [30 s of denaturation at 95 °C, 30 s of annealing at 52 °C; 30 s product amplification at 60 °C] and the reaction was set up according to the manufacturer’s protocol of the QuantiNova SYBR Green PCR Kit (# 208252, Qiagen). Next, 2 µl of PCR product reaction was subjected to a new cycle of amplification using the Amplitaq gold DNA Polymerase (# 4398813, Thermo Fisher) using the same set of primers and cycling conditions. PCR products obtained were gel purified and cloned into the TOPO-TA cloning vector system (TOPO TA Cloning Kit for Sequencing, Thermo Fisher Scientific, US, # K4575J10). Upon transformation and plating on LB-Agar plates, 100 bacterial colonies were selected for plasmid purification. Purified plasmid DNA were isolated from the bacteria using QIAprep® Spin Miniprep Kit (# 27106, Qiagen) and sequenced according to Sanger by Eurofins Scientific (Luxembourg) to determine number and position of C and U and depict the methylation pattern of the loci.

### RNA extraction, cDNA synthesis and qPCR

Total RNA was extracted from pancreas and testis (approximately 25-30 mg) according to the manufacturing protocol of RNeasy Plus Mini Kit (# 74136, Qiagen). 2 μL of total RNA was quantified by NANODROP 2000 spectrophotometer (Thermo Fisher). 2 μg of total RNA was converted into cDNA using SuperScript VILO Master Mix (# 11755050, Thermo Fisher) as indicated by the manufacturer and qPCR analysis was performed using QuantiNova SYBR PCR Master Mix (# 208252, Qiagen). The amplification was performed using the Real-Time PCR System StepOnePlus, Applied Biosystems (Thermo Fisher), with the following cycling conditions: 95 °C for 3 min followed by 40 cycles of [30 s of denaturation at 95 °C, 30 s of annealing at 52 °C; 30 s product amplification at 60 °C]. The relative gene expression analysis of target genes was conducted in comparison with the β-actin housekeeping control gene following the comparative 2^−∆Ct^ method.

### List of primers used

**Table:** 

Application	*target*	*strand*	*Sequence (5’-3’)*
transcript analysis	β-actin	Forward	CTACCTCATGAAGATCCTGACC
		Reverse	CACAGCTTCTCTTTGATGTCAC
	H19	Forward	AGAGGACAGAAGGGCAGTCA
		Reverse	TGGGTGGACAATTAGGTGGT
	IGF II	Forward	CGCTTCAGTTTGTCTGTTCG
		Reverse	AAGCAGCACTCTTCCAC
	STAR	Forward	TCAACTGGAAGCAACACTCTAT
		Reverse	ATCTTACTTAGCACTTCGTCCC
	CYP11A1	Forward	AGTATTATCAGAGGCCCATTGG
		Reverse	AACATCTGGTAGACAGCATTGA
	Hsd3β1	Forward	AAAGGTACCCAGAACCTATTGG
		Reverse	CTGTATGGGTATGGATCAGACC
	CYP17A1	Forward	GAGGTGAAGAGGAAGATCCAAA
		Reverse	ATACGAAGCACTTCTCGGATAG
	Hsd17b3	Forward	CGCCGATGAGTTTGTTAAAGAA
		Reverse	GGATCCGGTTCAGAATTATTGC
	CYP21A1	Forward	CAAGATGTGGTGGTGCTAAATT
		Reverse	GCCTTCCACATGAGAGAGTAAT
	CYP19A1 exon II	Forward	GTTCTTGGAAATGCTGAACC
		Reverse	CCAGACTCTCATGAACTCTC
	CYP19A1 ovary exon I	Forward	CACCCTTCCAAGTGACAGGA
		Reverse	AAAAAAGTAAAGTTCTATGGGAA
	CYP19A1 brain exon I	Forward	CTATCCGGTTTTTAAACGGC
		Reverse	GGATCTGCTGGTCACTTCTA
Methylation sensitive PCR	CYP19A1 brain exon I	Forward	CTATCCGGTTTTTAAACGGC
		Reverse	GGATCTGCTGGTCACTTCTA
	IGFII/H19 ICR	Forward	GGAACCGCCAACAAGAAAGT
		Reverse	GGTCTTTCCACTCACAACGG
gDNA amplification post-bisulfite	CYP19A1 brain exon I	Forward	CTATCYGGTTTTTAAAYGGC
		Reverse	GGATCTGCTGGTCACTTCTA
	IGFII/H19 ICR	Forward	TTTATTTATAAYGGTTTTTGTGTTTTTT
		Reverse	AAACCCCTAACTAACTAATTTATAACAAATAA

### Statistical analysis

Statistical analyses were performed using Prism 6 (GraphPad software). Comparisons between two groups were done by using the *t*-test, analysing each row individually, not assuming consistent standard deviation and when necessary, correcting for multiple comparison using the Holm-Sidak method. Statistical significance is expressed as *p* value, Cohen’s *d* value and the effect-size correlation. Difference with *p* values > 0.05 were considered non statistically significant. Cohen’s *d* value and the effect-size correlation, *r*, were measured using the means and standard deviations of two groups (treatment and control). Cohen’s *d* = *M*_eanTreatment_ – *M*_eanControl_/sd_pooled_ where sd_pooled_ = √[(sd_Treatment_^2^ + sd_Control_^2^)/2] and *r* = *d*/√(*d*^2^ + 4). Effect size was considered: *r* > 0.85 = large, 0.5 < *r* < 0.85 = medium, 0.2 < *r* < 0.5 = small, *r* < 0.2 = no effect.

## Results

**Fig. 1:**
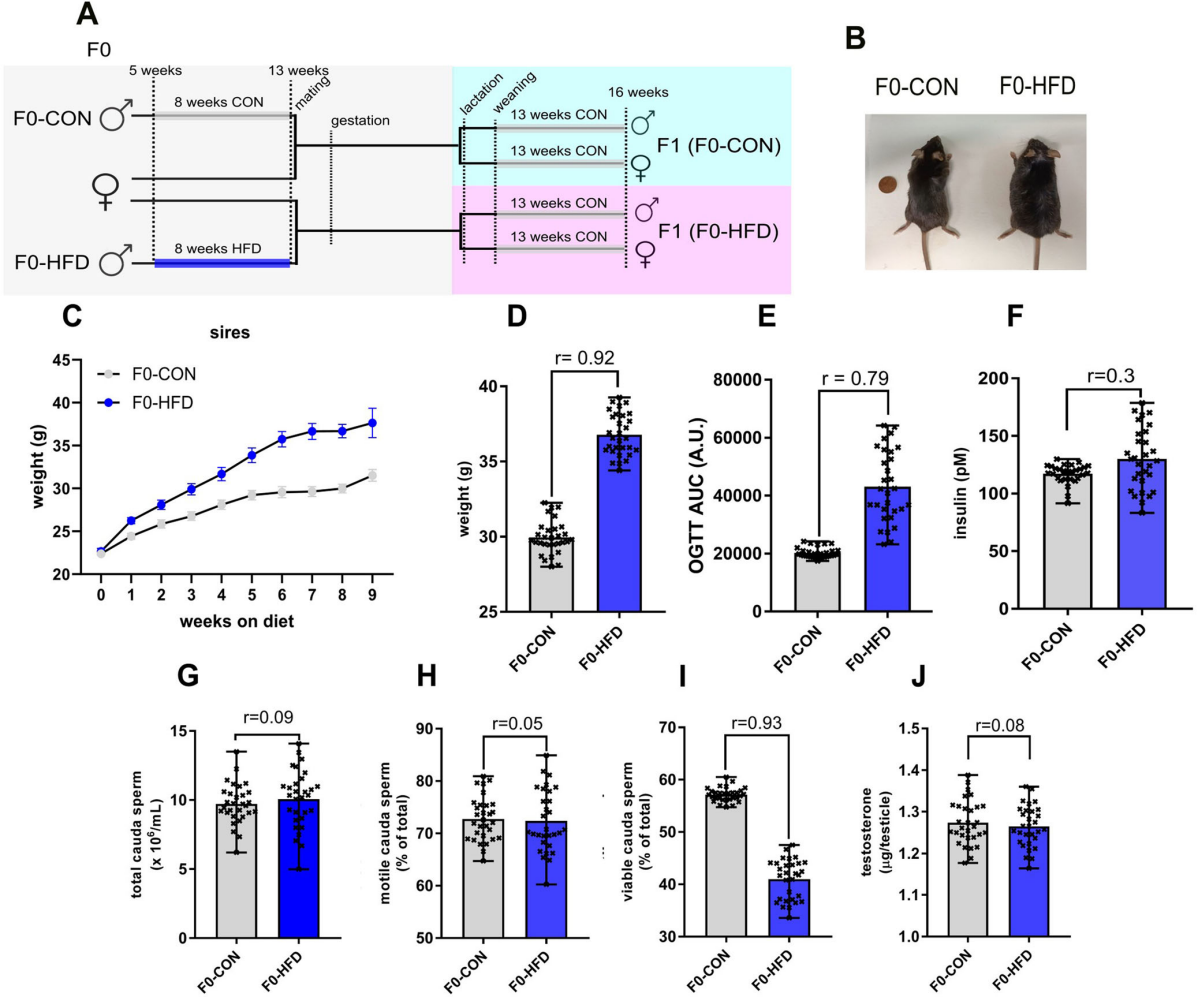
F0-HFD males present a mild diabetic phenotype with moderate body weight gain, fasting hyperglycaemia, and glucose intolerance, minimal alterations in sperm parameters, unaltered fertility, and testosterone levels. **A** Feeding and breeding scheme used in this study. Five weeks old C57BL/6 J males (F0) were fed either a control (CON) diet (F0-CON, *n* = 30) or high-fat (HFD) diet, (F0-HFD, *n* = 30) for eight weeks, to be then mated to inbred C57BL/6 J females fed a regular CON diet. F1 from F0-CON (F1(F0-CON), *n* = 43 per sex, each from a different mother) and F1 from F0-HFD (F1(F0-HFD), *n* = 43 per sex, each from a different mother), were fed a CON diet from weaning to week 17 to then undergo metabolic testing, biochemical analyses and post-mortem necropsy. **B**–**D** Representative picture of F0-CON and F0-HFD males at culling (**B**). Body weight, weekly gain (**C**) and at necropsy (**D**). **E**, **F** Compared to F0-CON, F0-HFD males present increased OGTT area under the curve (AUC) (**E**) and moderate increase in insulinemia (**F**). **G**–**I** Concentration of sperm cells expressed as number of sperms/mL counted in caput epididymis in F0-HFD compared to F0-CON (**G**). Sperm viability expressed as % of viable sperm cells in caput epididymis in F0-HFD compared to F0-CON sires (**H**). Sperm motility expressed as % motile sperm cells in *cauda epididymis* of F0-HFD compared to F0-CON sires (**I**). **J** Testicular testosterone levels (μg/testicle) measured in F0-HFD testis compared to F0-CON. In **C**, data are presented as mean and SEM (*n* = 30). In **D**–**J**, individual data are scattered as dark crosses together with their mean and range. Bars are labelled with effect size: *r* > 0.85 = large, 0.5 < *r* < 0.85 = medium, 0.2 < *r* < 0.5 = small, *r* < 0.2 = no effect.

To give F0 sires an obesogenic stimulus without affecting their fertility, we fed C57BL/6 J inbred males HFD (60% of calories from fat, see methods for details) for 8 weeks (Fig. 1A). The length of the treatment was long enough to affect an entire cycle of spermatogenesis (a process taking 35 days in mice), without causing severe reduction in mating efficiency or fertility rate.

### Fathers fed HFD predispose male progeny to hypoandrogenism and diabetes, and female progeny to improved glucose tolerance

As shown in Table 1 and Fig. 1 B–D, after 8 weeks of HFD and compared to F0 mice fed CON (F0-CON group, *n* = 30), F0 mice fed HFD (F0-HFD group, *n* = 30) present increased body weight at sacrifice, (F0-HFD:36.7 ± 1.4 g; F0-CON:30.0 ± 1.1 g, mean ± SD, effect size *r* = 0.92, *p* < 0.001) mostly due to epididymal fat and subcutaneous white adipose fat expansion (Table 1 and Supplementary Fig. 1), and a mild diabetic phenotype with fasting hyperglycaemia (F0-HFD:106 ± 7 mg/dL; F0-CON: 83 ± 4 mg/dL, *r* = 0.89, *p* < 0.001) (Table 1), and reduced glucose sensitivity as shown by the oral glucose tolerance (OGTT) test (F0-HFD: 43095 ± 12335; F0-CON: 20276 ± 1752, *r* = 0.79, *p* < 0.001) Fig. 1E). However, F0-HFD sires present very mild hyperinsulinemia (F0-HFD: 130.1 ± 26.4; F0-CON: 117.3 ± 8.18 pM, *r* = 0.3, *p* = 0.14), (Fig. 1F), and still possess physiological cholesterol levels and liver functionality, as shown by triglycerides levels, HDL, LDL, ALT and AST, that are not significantly different from the control F0-CON group (Table 1). This mild pre-diabetic phenotype was also confirmed by WB analysis performed on liver homogenates of F0-HFD animals. Indeed, F0-HFD mice showed levels of phosphorylated AKT and AMPK comparable to those measured in sires of the control F0-CON group (Supplementary Figs. 2 and 3).

**Table 1:** Morphometric analysis (whole body, main organs and reproductive tract), hematochemical analysis, sperm analysis, sperm ROS staining (staining with DCF-DA or Mito Tracker CMX-ROS), glutathione (GSH) quantitation (TMR-IAA staining) and fertility rate assessment performed on adult F0-CON and F0-HFD

	F0-CON	F0-HFD	Effect size (*r*)	*p* Value (*r*)
Fathers (F0 generation)	*n* = 30	*n* = 30		
Body weight at sacrifice (g),	30.0 ± 1.1	36.7 ± 1.4	0.92	<0.001
Energy intake (kcal/mice/day)	9.5 ± 0.5	11.6 ± 1.2	0.75	<0.001
*Organ weight*				
Liver (g)	1.23 ± 0.28	1.33 ± 0.22	0.19	0.13
Testicle (g)	0.053 ± 0.028	0.051 ± 0.022	0.04	0.76
Prostate (g)	0.060 ± 0.044	0.057 ± 0.033	0.04	0.77
Seminal vesicles (g)	0.24 ± 0.09	0.26 ± 0.02	0.15	0.24
Abdominal fat (g/cm^3^)	0.11 ± 0.02	0.22 ± 0.03	0.90	<0.001
*Epidydimal fat (g)*				
Distal	0.123 ± 0.050	0.294 ± 0.105	0.72	<0.001
Central	0.062 ± 0.028	0.179 ± 0.105	0.60	<0.001
Proximal	0.036 ± 0.022	0.102 ± 0.061	0.58	<0.001
Caudal	0.007 ± 0.006	0.012 ± 0.006	0.38	0.002
*Clinical biochemistry*				
Glucose (mg/dL)	83 ± 4	106 ± 7	0.89	<0.001
Insulin (pM)	117.3 ± 8.18	130.1 ± 26.4	0.30	0.14
OGTT (AUC)	20276 ± 1752	43095 ± 12335	0.79	<0.001
Cholesterol total (mg/dL)	92.4 ± 12.7	110.39 ± 23.5	0.43	<0.001
Triglycerides (mg/dL)	139.2 ± 15.8	160.9 ± 20.2	0.51	<0.001
HDL (mg/dL)	43.2 ± 6.1	39.6 ± 3.7	0.33	0.008
LDL (mg/dL)	11.9 ± 0.9	8.9 ± 0.8	0.86	<0.001
ALT (U/L)	31.3 ± 2.1	33.2 ± 2.0	0.42	<0.001
AST (U/L)	51.2 ± 5.0	53.3 ± 6.0	0.18	0.15
*Sperm parameters*				
Concentration (×10^6^/mL)	9.7 ± 1.5	10.1 ± 2.1	0.09	0.40
Motility (caudal, %)	72.8 ± 3.7	72.2 ± 6.5	0.05	0.67
Viability (%)	57.1 ± 1.3	41.0 ± 3.8	0.93	<0.001
*ROS (DCF-DA staining) (% positive on total)*				
Total spermatozoa	13 ± 2	22 ± 2	0.91	<0.001
Caput spermatozoa	24 ± 2	57 ± 2	0.99	<0.001
Corpus spermatozoa	17 ± 2	28 ± 3	0.90	<0.001
Caudal spermatozoa	11 ± 2	19 ± 2	0.89	<0.001
*Mitochondrial ROS (Mito Tracker CMX-ROS, FI)*				
Caudal spermatozoa	7937 ± 1092	8843 ± 1320	0.35	0.005
Glutathione (fluorescence-TMR-IAA)				
Caudal spermatozoa	674 ± 49	683 ± 49	0.09	0.48
Fertility rate (%)	100	100		
Average number of pups	6 ± 0.2	5.8 ± 0.2	0.44	<0.001

Results are reported as mean ± SD (*n* = 30 per group). Effect size and *p* values are expressed in last columns as *r* and *p* values, respectively. Effect size: *r* > 0.85 = large, 0.5 < *r* < 0.85 = medium, 0.2 < *r* < 0.5 = small, *r* < 0.2 = no effect. Differences with *p* value > 0.05 were considered non statistically different.

HFD sires do not present change in liver, testis, prostate and seminal vesicle weight and gross morphology (Supplementary Fig. 1). However, while sperm count and motility are not altered by HFD, sperm viability is reduced in the F0-HFD group (F0-HFD:41.0 ± 3.8%; F0-CON:57.1 ± 1.3%, *r* = 0.93, *p* < 0.001) (Table 1 and Fig. 1H–I). Loss of viability is probably due to spermatic oxidative stress induced by the HFD (especially in *caput* sperm) where we measured an increased percentage of sperm cells positive to di-cholo-fluorescein (DCF-DA) (F0-HFD:57 ± 2% %; F0-CON:24 ± 2%, *r* = 0.99, *p* < 0.001) (Table 1). Increased ROS production in F0-HFD spermatozoa was not mitochondrial-derived as shown by the fluorescence of the mitochondrial ROS probe Mitotracker CMX- ROS, for which the difference in intensity in *cauda* sperm between F0-HFD and F0-CON groups was not statistically different (Table 1). Oxidative stress induced by the HFD did not manifest as reduction in GSH measured in *cauda* sperm as measured by the probe TMR-IAA (Table 1). The dysmetabolic phenotype of HFD sires does not affect their testicular testosterone level that results not statistically different between HFD and CON groups (F0-HFD:1.265 ± 0.05; F0-CON:1.273 ± 0.05, *r* = 0.08, *p* = 0.54) (Fig. 1J).

After 8 weeks of HFD or control diet, 13-week-old F0 males were mated to C57BL/6 J inbred females (*n* = 120). The F0 mothers chosen for mating were nulliparous, non-diabetic and fed a control diet, from weaning to mating (13 weeks) (see Supplementary Table 1). The standard diet was maintained after mating (verified by vaginal plug) and during gestation and lactation. Male and female F0 interacted only at mating, after which F0 sires were returned to their cages. The dysmetabolic phenotype of HFD fathers did not affect fertility rate, number of pups per litter, offspring sex ratio, average weight of the pups at birth and at reduction, all resulting not statistical different between HFD and CON groups (see Supplementary Table 1). Mating of the F0 generation led to an equal number of litters for each of the two groups of fathers. In order to avoid undernourishment phenomena, which often occurs in overpopulated litter and is known to represent an epigenetic environmental stimulus for the progeny^29^, the number of pups per litter was reduced to 5. Pups removed were chosen randomly. After weaning, one female and one male pup from each litter were randomly selected and allocated into two new groups: F1 generation from F0-HFD was named F1(F0-HFD) (*n* = 43 per sex) while the generation from F0-CON was named F1(F0-CON) (*n* = 43 per sex) (Fig. 1A and Supplementary Table 1). Independently from their fathers, male and female offspring were fed CON diet from weaning up to week 16, when all underwent metabolic testing, biochemical analyses, and post-mortem necropsy.

**Fig. 2:**
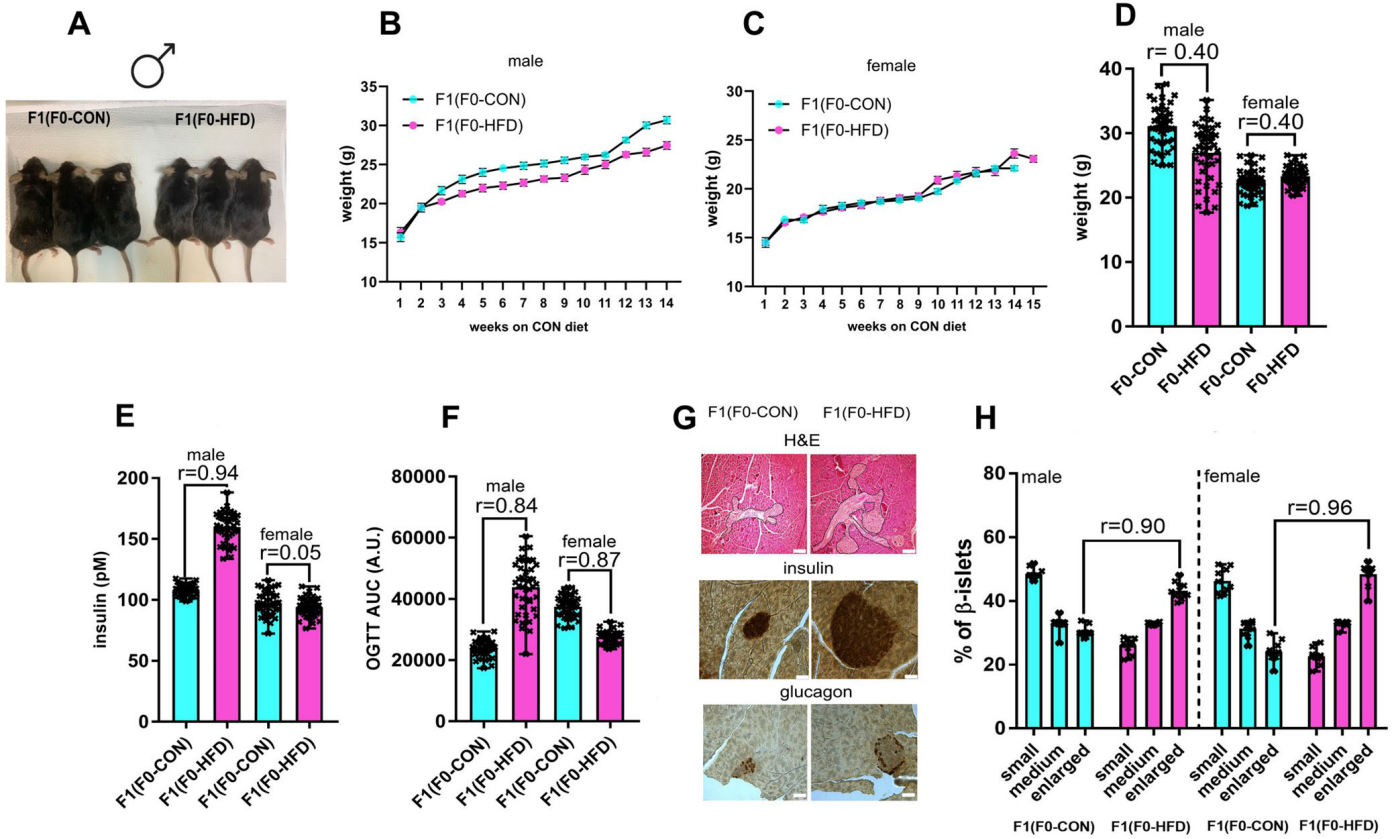
F0-HFD sires predispose male offspring to hyperinsulinemia, hyperplasia of pancreatic β-cells, insulin resistance and altered expression of *IGF-II* and *H19.* **A**–**D** Compared to F1(F0-CON), F1(F0-HFD) males present decreased weekly body weight gain (**B**) and at necropsy (**A**, **D**). The effect is dimorphic since compared to F1(F0-CON), F1(F0-HFD) females present unaltered weekly body weight gain (**C**) and moderate weight gain at necropsy (**D**). **E** Compared to male F1(F0-CON), male F1(F0-HFD) present fasting hyperinsulinemia. F1(F0-HFD) females present insulinemia comparable to F1(F-CON) females. **F** Compared to male F1(F0-CON), male F1(F0-HFD) present glucose resistance as shown by the increased OGTT test AUC. F1(F0-HFD) females present reduced OGTT AUC compared to F1(F0-CON) females. **G** Representative immunohistochemical staining of pancreatic islets of F1(F0-HFD) and F1(F0-CON) males revealing expansion of insulin positive pancreatic β-cell islets [Hematoxylin and Eosin (upper panels, pancreatic islets are indicated by dashed lines) or anti-Insulin (middle panels) and α-glucagon (lower panels) antibody staining (Scale bar: 75 μm)]. **H** Quantification of enlarged, medium and small pancreatic islets (expressed as percentage of total number of islets) showing an increased number of large islets in pancreas of male and female F1(F0-HFD) compared to F1(F0-CON). For **B**–**F**, (*n* = 43 per sex, each from a different mother). For **H**, (*n* = 10 per sex, each from a different mother). In **B**, **C** data are presented as mean and SEM. In **D**–**H**, individual data are scattered as dark crosses together with their mean and range. Bars are labelled with effect size: *r* > 0.85 = large, 0.5 < *r* < 0.85 = medium, 0.2 < *r* < 0.5 = small, *r* < 0.2 = no effect.

As shown in Table 2 and Fig. 2A–C, growth differences between F1(F0-CON) and F1(F0-HFD) progeny manifested starting 3 weeks post-weaning, with F1(F0-HFD) male presenting reduced body weight compared to F1(F0-CON) male (Fig. 2 panels B, D). Body weight difference was maintained until culling, with F1(F0-HFD) male being leaner than F1(F0-CON) male (F0-HFD: 27.4 ± 4.3; F0-CON: 30.7 ± 3.2 g, *r* = 0.40, *p* < 0.001) (Fig. 2 panels B, C). The reduced body weight was not due to a different food intake, which appeared statistically similar between the two groups (Table 2). However, despite the difference, both F1(F0-HFD) and F1(F0-CON) average body weights fall in the expected weight range of the mouse strain^30^, suggesting that the leaner phenotype of F1(F0-CON) might be still considered physiologic and not linked to pathology. The leaner phenotype manifests only in the male F1(F0-HFD) progeny. The body weight of F1(F0-HFD) female was slightly increased compared to F1(F0-CON) (F1(F0-HFD): 23.6 ± 1.5; F1(F0-CON): 22.1 ± 1.9 g, *r* = 0.40, *p* < 0.001), suggesting a dimorphic influence of F0-HFD fathers on the progeny (Fig. 2 panels C, D). Sex differences in the intergenerational inheritance of metabolic traits have been demonstrated in humans and in many animal models^31^.

**Table 2:** Morphometric (whole body and pancreas) analysis, hematochemical analysis, glycemia, insulinemia, OGTT (AUC), testosterone levels of male and female adult F1(F0-CON) and F1(F0-HFD). Results are reported as mean ± SD (*n* = 43 per group, per sex, each from a different mother Effect size and *p* values are expressed in last columns as *r* and *p* values, respectively

	F1(F0-CON)	F1(F0-HFD)	Effect size (r)	p value (r)
Male F1	43	43		
Body Weight at sacrifice(g)	30.7 ± 3.2	27.4 ± 4.3	0.40	<0.001
Food Intake (g/mice/day)	3.9 ± 0.2	3.9 ± 0.4	0	>0.99
*Clinical biochemistry*				
Glucose (mg/dL)	113.4 ± 7.2	106.0 ± 3.0	0.55	<0.001
Insulin (pM)	106 ± 5	160 ± 12	0.94	<0.001
OGTT (AUC)	23425 ± 2821	43569 ± 8680	0.84	<0.001
Cholesterol total (mg/dL)	96.3 ± 15.1	93.7 ± 11.8	0.09	0.38
Triglycerides (mg/dL)	150.6 ± 17.3	144.9 ± 18.3	0.15	0.14
HDL (mg/dL)	45.6 ± 5.2	43.7 ± 7.8	0.14	0.19
LDL (mg/dL)	9.3 ± 1.2	8.7 ± 1.3	0.23	0.04
ALT (U/L)	34.2 ± 1.8	32.5 ± 2.3	0.38	<0.001
AST (U/L)	50.6 ± 3.2	52.2 ± 4.1	0.21	0.04
Testosterone (µg/testicle)	1.242 ± 0.062	1.169 ± 0.035	0.75	<0.001
*Pancreatic islets*				
% Small (0–5000 μm^2^)	48.5 ± 1.8	26.1 ± 2.4	0.98	<0.001
% Medium (5001–10,000 μm^2^)	32.8 ± 2.4	32.9 ± 2.3	0.02	0.85
% Large (>10,000 μm^2^)	30.4 ± 1.7	43.2 ± 2.6	0.95	<0.001
Female F1	43	43		
Body weight at sacrifice (g)	22.1 ± 1.9	23.6 ± 1.5	0.40	<0.001
Food intake (g/mice/day)	3.8 ± 0.2	3.7 ± 0.7	0.09	0.37
*Clinical biochemistry*				
Glucose (mg/dL)	102.0 ± 4.3	123.0 ± 7.4	0.86	<0.001
Insulin	96.0 ± 10.0	95.1 ± 9.2	0.05	0.67
OGTT (AUC)	36873 ± 3253	27546 ± 1952	0.87	<0.001
Cholesterol total (mg/dL)	92.0 ± 11.2	90.4 ± 9.3	0.08	0.47
Triglycerides (mg/dL)	122.7 ± 10.1	124.9 ± 11.4	0.10	0.35
HDL (mg/dL)	42.6 ± 3.5	41.6 ± 4.9	0.11	0.28
LDL (mg/dL)	11.4 ± 2.2	10.9 ± 1.9	0.12	0.26
ALT (U/L)	32.4 ± 1.1	30.6 ± 1.7	0.53	<0.001
AST (U/L)	51.7 ± 2.3	50.2 ± 3.7	0.23	0.03
*Pancreatic islets*				
% Small (0–5000 μm^2^)	46.2 ± 3.8	22.6 ± 2.5	0.96	<0.001
% Medium (5001–10,000 μm^2^)	31.3 ± 2.5	32.8 ± 1.0	0.36	<0.001
% Large (>10,000 μm^2^)	24.1 ± 3.3	48.4 ± 3.5	0.96	<0.001

Effect size: *r* > 0.85 = large, 0.5 < *r* < 0.85 = medium, 0.2 < *r* < 0.5 = small, *r* < 0.2 = no effect. Differences with *p* value > 0.05 were considered non statistically different.

Despite fed control diet, F1(F0-HFD) male presented a severe diabetic phenotype with fasting hyperinsulinemia (F1(F0-HFD): 160 ± 12 pM; F1(F0-CON): 106 ± 5 pM, *r* = 0.94, *p* < 0.001) and glucose resistance as shown by the OGTT (F1(F0-HFD): 43569 ± 8680 AUC; F1(F0-CON): 23425 ± 2821 AUC; *r* = 0.84, *p* < 0.001) (Table 2 and Fig. 2E, F). As for weight gain, the diabetic phenotype was sexually dimorphic, with F1(F0-HFD) female presenting fasting insulin levels not different from F1(F0-CON) group (Table 2 and Fig. 2E, F), and, intriguingly, augmented performance in the OGTT (F1(F0-HFD): 27546 ± 1952 AUC; F1(F0-CON): 36873 ± 3253 AUC, *r* = 0.87, *p* < 0.001) (Fig. 2F). A likely reason of the fasting hyperinsulinemia measured in F1(F0-HFD) male mice, could have been pancreatic β-cell expansion. Indeed, immuno-histological analysis of pancreatic tissues of F1(F0-HFD) confirmed an increased percentage of enlarged pancreatic islets (F1(F0-HFD): 43.2 ± 2.6%; F1(F0-CON): 30.4 ± 1.7%, *r* = 0.95, *p* < 0.001) (Table 2 and Fig. 2G, H). Enlarged islets presented increased number of insulin-positive pancreatic β-cell cells but not of glucagon-positive α-cell (Fig. 2G). Intriguingly, pancreatic β-cell expansion occurs also in F1(F0-HFD) female, where we as well measured an increased percentage of enlarged pancreatic islets (F1(F0-HFD): 48.4 ± 3.5; F1(F0-CON): 24.1 ± 3.3%, *r* = 0.96, *p* < 0.001) (Table 2 and Fig. 2 panel H). Despite the diabetic phenotype, liver functionality in male F1(F0-HFD) is similar to F1(F0-CON), as shown by circulating Triglycerides, HDL, LDL levels, ALT and AST activities, which are not significantly different from the control group. F1(F0-HFD) do not present gross change in testicle, prostate and seminal vesicle weight and morphology (Table 2).

We envisaged that one of the likely reasons of this dimorphic phenotype promoted by paternal F0-HFD could have been alteration of testosterone levels. During development, testosterone, is important for body weight gain especially in male mice. Similarly, testosterone effect on glucose metabolism has been shown to be dimorphic^32^. Reduced testosterone levels promote diabetes in male mice and human. On the contrary, increased testosterone levels promote diabetes in females^33^.

In order to verify if testosterone was altered in F1(F0-HFD) males, we measured its levels in the testis of male offspring. Indeed, F1(F0-HFD) males presented reduced levels of testis testosterone compared to F1(F0-CON) males (F1(F0-HFD): 1.169 ± 0.035 µg/testicle; F1(F0-CON): 1.242 ± 0.062 µg/testicle, *r* = 0.75, *p* < 0.001) (Table 2).

### Fathers fed HFD influence the methylation status and activity of aromatase in offspring testis

**Fig. 3:**
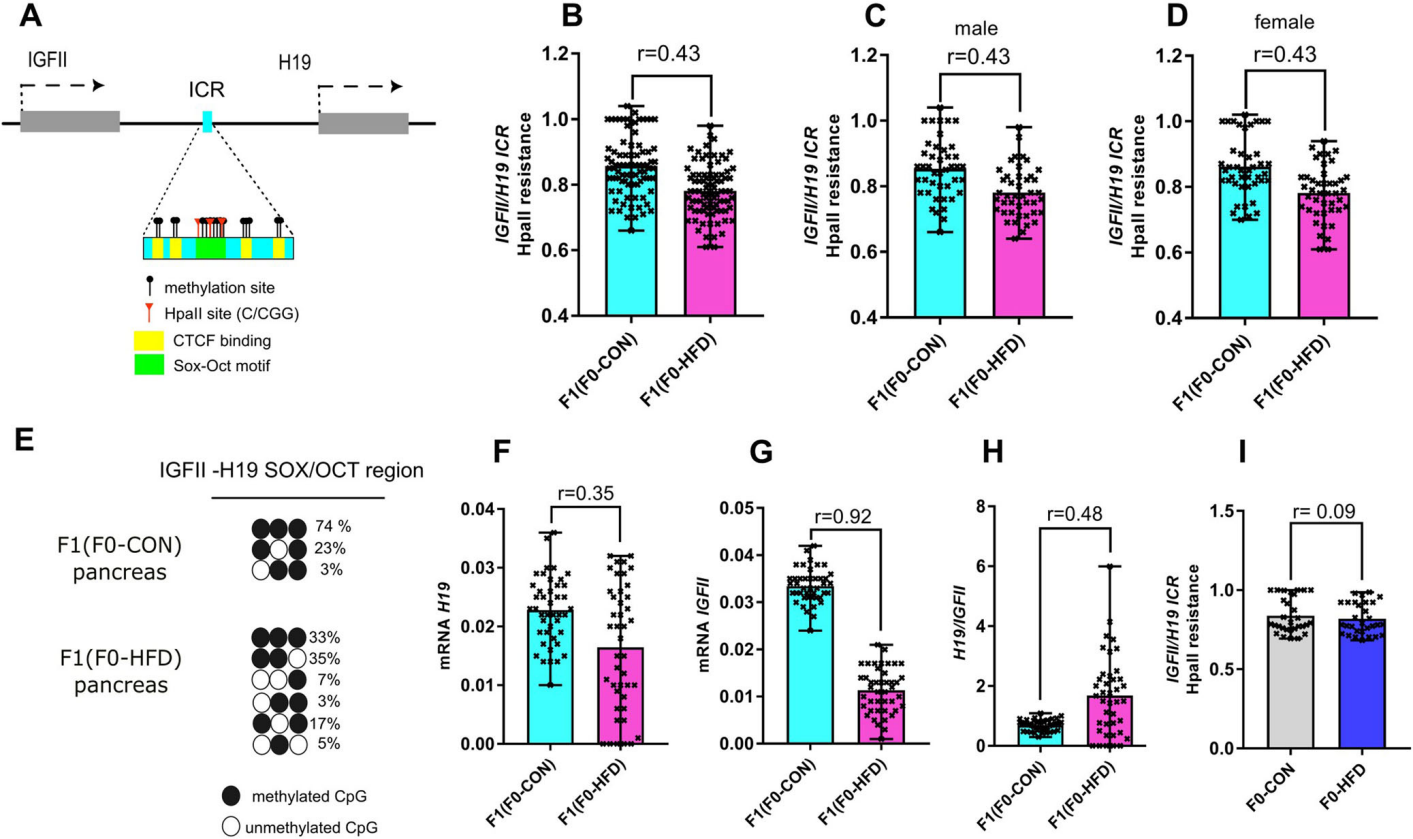
F0-HFD sires predispose male offspring to altered methylation pattern at the *IGF-II* and *H19* enhancer region. **A** Representative scheme of *IGF-II/H19* enhancer region on mouse chromosome VII. The scheme illustrates the distribution of CpG cytosines and HpaII (C/CGG) restriction sites in the ICR, as well as the CTCF biding sites (yellow) and the Sox-Oct motif (light green); **B**–**D** Methylation sensitive qPCR analysis of gDNA extracted from pancreas of male (**C**) and female (**D**) F1(F0-HFD) and F1(F0-CON) revealing significant reduction in methylation levels of pancreatic ICR region in F1(F0-HFD) compared to F1 (F0-CON), in male F1(F0-HFD). (For analysis in (**B**–**D**), fold change is expressed as 2^−∆ct^ vs undigested gDNA). **E** Sanger sequencing of bisulfite converted pancreatic gDNA of F1(F0-HFD) and F1(F0-CON). The analysis confirmed that CpGs allocated in one of the subregion of the SOX/OCT binding site of the ICR in F1(F0-HFD) males present a reduced pattern of methylation compared to F1(F0-CON) males. **F**–**H** Quantitation of total (**F**, **G**) and relative (**H**) IGFII and H19 transcripts performed by RT-qPCR analysis in pancreas of F1(F0-HFD) and F1 (F0-CON) and revealing decreased transcription of both genes in F1(F0-HFD) males compared to F1(F0-CON) males. **I** Methylation sensitive qPCR analysis of gDNA extracted from pancreas of F0-HFD and F0-CON sires revealing unaltered methylation levels of pancreatic ICR region among the two groups. (For analysis in B–D, fold change is expressed as 2^-∆ct^ vs undigested gDNA). For analysis in **F**–**H**, fold change is expressed as 2^−∆ct^ vs housekeeping gene *β-actin*. For analysis in **B**–**H**, comparisons were made using a *t* test (*n* = 43 per sex, each from a different mother). For analysis in **I**, comparisons were made using a *t* test (*n* = 30 per group). Individual data are scattered as dark crosses together with their mean and range. Bars are labelled with effect size: *r* > 0.85 = large, 0.5 < *r* < 0.85 = medium, 0.2 < *r* < 0.5 = small, *r* < 0.2 = no effect.

As a positive control of epigenetic signalling impacting F1(F0-HFD) offspring metabolic phenotype, we analysed the methylation status of the enhancer region allocated between the *IGF-II* and the *H19* genes, on mouse chromosome VII^34,35^. This region is epigenetically regulated by the presence of several DMRs and one ICR, that is known to bind the transcriptional repressor CTCF and the transcription factors OCTs and SOXs (Fig. 3A)^34,35^. The methylation status of this enhancer can influence the selective expression of the *IGFII* gene to the detriment of the long non-coding RNA encoded by the downstream gene *H19*^36^. As shown in Fig. 3 panels B–D, methylation sensitive qPCR of gDNA extracted from pancreas of F1(F0-HFD) male revealed reduced methylation in the SOX/OCT binding region (408 bp of Chr VII 142,134,647-142,135,313) (F1(F0-HFD): 0.78 ± 0.08; F1(F0-CON): 0.85 ± 0.09 (2^−Δct^ vs undigested gDNA), *r* = 0.43). This reduction in methylation occurred in both male (Fig. 3C, *r* = 0.43, p < 0.001) and female offspring (Fig. 3D*r* = 0.43, *p* < 0.001). Sanger sequencing of bisulfite converted pancreatic gDNA confirmed that cytosines allocated in a subregion of the SOX/OCT binding site of the ICR were statistically less methylated in pancreatic cells of F1(F0-HFD) males compared to F1(F0-CON) males, confirming specific epigenetic differences occurring in F1(F0-HFD) progeny (Fig. 3E). *H19* and *IGFII* transcripts are barely expressed in adult pancreas as shown by their difference in expression compared to the housekeeping gene *β-actin*. Notwithstanding, in the pancreas of F1(F0-HFD) both *H19* (F1(F0-HFD): 0.017 ± 0.010; F1(F0-CON): 0.024 ± 0.005, 2^−^^ΔCt^ vs *β-actin*, *r* = 0.35, *p* < 0.001) and *IGFII* (F1(F0-HFD): 0.011 ± 0.005; F1(F0-CON): 0.034 ± 0.004; 2^−^^ΔCt^ vs *β-actin* *r* = 0.92, *p* < 0.001) were less transcribed compared to F1(F0-CON) with a ratio between *H19* and *IGFII* transcripts increasing around three times and confirming selective expression of *H19* transcript to the detriment of IGF-II (Fig. 3F–H). Interesting, the methylation status of ICR was statistically similar in F0-CON and F0-HFD testis, suggesting that epigenetic changes occurred upon egg fertilisation (F0-HFD: 0.78 ± 0.08; F0-CON: 0.85 ± 0.09 (2^−Δct^ vs undigested gDNA), *r* = 0.03, *p* = 0.002) (Fig. 3I).

**Fig. 4:**
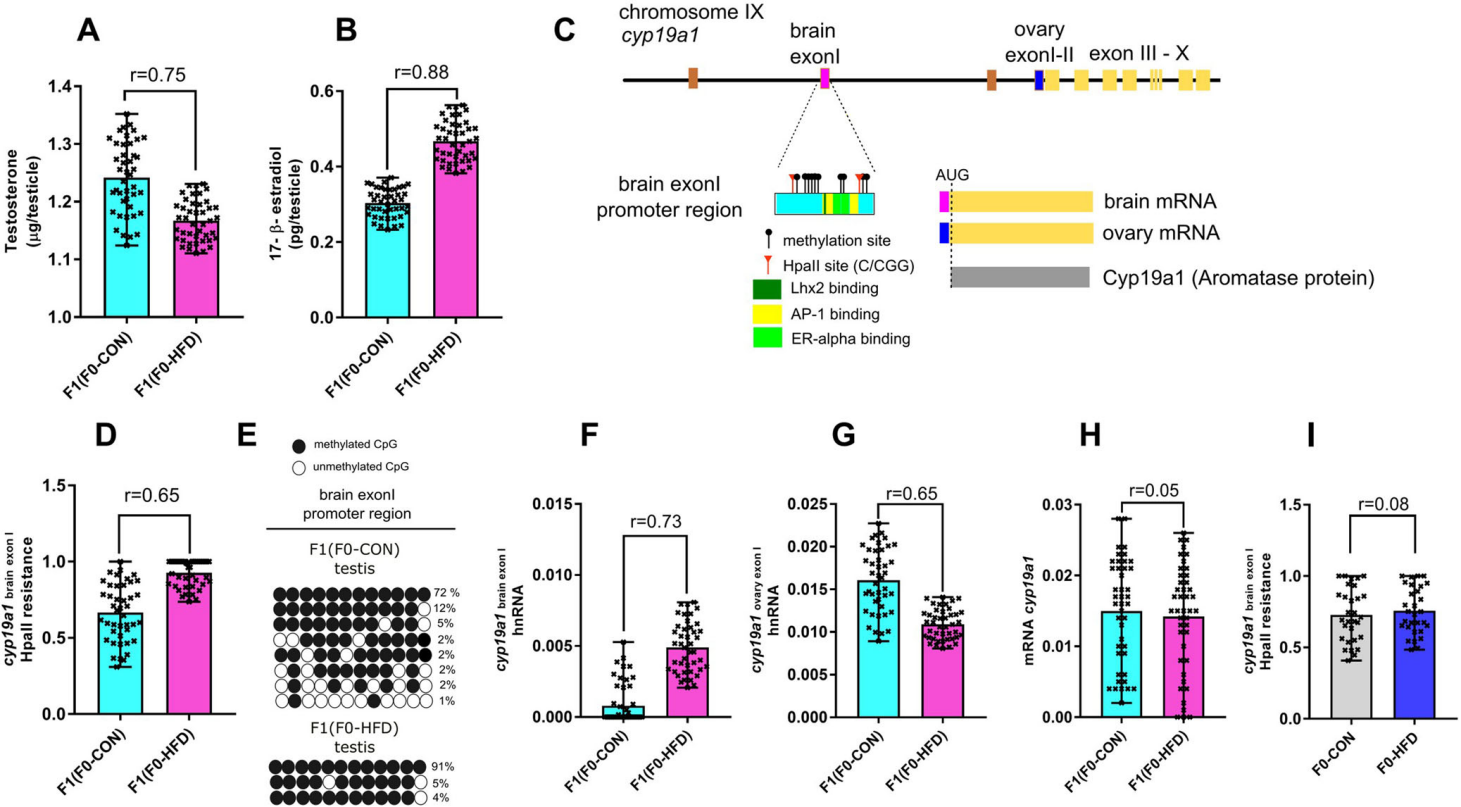
F0-HFD sires influence the methylation status of *cyp19a1* in offspring testis affecting its transcription and alternative splicing, and predispose male progeny to hypoandrogenism. **A**, **B** Testicular testosterone and 17-β estradiol levels (μg and pg /testicle, respectively) measured in F1(F0-HFD) testis compared to F1(F0-CON). F1(F0-HFD) present decreased testosterone and increased 17-β estradiol levels compared to F1(F0-CON). **C** Representative scheme of *cyp19a1* gene locus. Murine *cyp19a1* gene is located on chromosome IX. Nine translated exons (exons II–X) are preceded by several untranslated exons I (for simplicity the cartoon focuses only on brain and ovary exonI) each controlling the transcription of different hnRNAs. Primary sequence of the Aromatase protein is not affected by the different hnRNAs. The scheme illustrates distribution of CpG cytosines and HpaII (C/CGG) restriction sites in the region corresponding to brain exon I: Oestrogen Receptor (pale yellow), Jun/Fos (AP1 light green) and Lhx2 binding site (dark green). **D** Methylation sensitive qPCR analysis of gDNA extracted from testis of F1(F0-HFD) and F1(F0-CON) revealed significant increase in CpG methylation levels at brain exon I region (300 bp, minus strand of Chr IX: 54,136,106-54,136,406) in F1(F0-HFD) compared to F1 (F0-CON). **E** Sanger sequencing of bisulphite converted testis gDNA of F1(F0-HFD) and F1(F0-CON). The analysis confirmed increased methylation of cytosines in brain exon I of F1(F0-HFD) testis compared to F1(F0-CON). **F**–**H** Quantitation of brain, ovary and total hnRNAs of *cyp19a1* performed by RT-qPCR analysis in testis of F1(F0-HFD) and F1(F0-CON) and revealing preferential transcription of brain hnRNA splicing isoform over the ovary hnRNA and unaltered total levels of *cyp19a1* transcription. For analysis in **D**, fold change is expressed as 2^−∆ct^ vs undigested gDNA. **I** Methylation sensitive qPCR analysis of gDNA extracted from pancreas of F0-HFD and F0-CON sires revealing unaltered methylation levels of *cyp19a1* brain exon I between the two groups. For analysis in **F**–**H** fold change is expressed as 2^−∆ct^ vs housekeeping gene *β-actin*. For each analysis *n* = 43 per sex, each pup from a different mother. For analysis in **I**, *n* = 30 per group. Individual data are scattered as dark crosses together with their mean and range. Bars are labelled with effect size: *r* > 0.85 = large, 0.5 < *r* < 0.85 = medium, 0.2 < *r* < 0.5 = small, *r* < 0.2 = no effect.

In order to identify new genetic regions repositories and/or transducers of epigenetic inheritance and inspired by the metabolic phenotype of the F1(F0-HFD) offspring we focused on genes involved in testosterone homoeostasis. Decreased testicular testosterone is most often associated with increased testosterone conversion into 17-β estradiol by the enzyme Aromatase, encoded by the *cyp19a1* gene. We therefore measured testicular 17-β estradiol levels in F1 offspring. As shown in Fig. 4A, B, reduced testosterone levels correspond to increased 17-β estradiol levels in F1(F0-HFD) testis compared to F1(F0-CON) males (F1(F0-HFD): 0.47 ± 0.06; F1(F0-CON): 0.31 ± 0.04 pg/testicle, *r* = 0.88, *p* < 0.001), suggesting increased intratesticular aromatase activity. The murine *cyp19a1* gene is located on chromosome IX. Nine translated exons (exons II–X) are preceded by different exons I, (with brain and ovary exon I being the best characterised and described) (Fig. 4C)^37^. Different promoters control the transcription of different heterogenous nuclear RNA (hnRNAs) each including one or more exon I. These pre-mRNAs undergo alternative splicing to generate specific mRNAs presenting only one of the different exons I^37,38^. Although these alternative exons I, the different mature mRNAs do not alter the primary sequence of the Aromatase protein, since the first translated codon is located in the middle of exon II. Most of the tissues express only one of the different hnRNAs. On the contrary, testis expresses both brain, and ovary exon-I containing variants. Compared to other genetic loci and considering the low frequency of cytosines, the region cannot be considered a CpG island. To verify whether F0-HFD sire might have imposed a change in the methylation status at the promoter regions of the aromatase gene in their progeny, we analysed fluctuations in cytosine methylation in the 5’ region of brain exon I, the only one shown to be epigenetically controlled, at least in glia cells^39^. As shown in Fig. 4C, the brain promoter/exon I region of *cy19a1* contains 12 isolated CpGs, all surrounded by DNA regulatory elements including oestrogen receptor (ER)^40^, Jun/Fos^41^ and Lhx2^42^ binding sites. As shown in Fig. 4D, methylation sensitive qPCR of gDNA extracted from the testis of F1(F0-HFD) male revealed increased methylation in the promoter/exon I region (300 bp, − strand of Chr IX: *54,136,106-54,136,406*) (F1(F0-HFD): 0.93 ± 0.09 (2^−^^Δct^ vs undigested gDNA); F1(F0-CON): 0.66 ± 0.19 (2^−Δct^ vs undigested gDNA), *r* = 0.65, *p* < 0.001). Sanger sequencing of bisulfite converted testis gDNA (Fig. 4E) confirmed that cytosines allocated in the promoter region of brain exon I were more methylated in F1(F0-HFD) males compared to F1(F0-CON), confirming specific epigenetic signalling affecting HFD-F1 male progeny also in this region.

As shown by the analysis of the transcripts by qPCR, this alteration in the methylation status affected the alternative hnRNAs (Fig. 4F–H). While hnRNA of *cyp19a1* presenting the ovary exon I is predominant in F1(F0-CON) testis (F1(F0-HFD): 0.011 ± 0.002; F1(F0-CON): 0.016 ± 0.004 2^(−ΔCt)^), *r* = 0.65, *p* < 0.001) the version of hnRNA containing brain exon I is predominant in F1(FO-HFD), (F1(F0-HFD): 0.005 ± 0.002; F1(F0-CON): 0.0008 ± 0.0019 2^(−ΔCt)^, *r* = 0.73, *p* < 0.001) suggesting that F0-HFD fathers, at least in offspring testis, might have predisposed offspring to a specific hnRNA isoform and thus to an alternative spliced mRNA variant. Interestingly, this change does not affect the overall levels of exon II transcript, which instead remains statistically unchanged among the groups, suggesting that the switch between the two hnRNA variants do not influence stability or half-life of the transcript. Again, as seen for the methylation status of ICR, the methylation status of in the promoter/exon I region of *cy19a1* was statistically similar in F0-CON and F0-HFD testis, suggesting that epigenetic changes at this locus occurred upon egg fertilisation ((F0-HFD: 0.76 ± 0.16 (2^−Δct^ vs undigested gDNA); F0-CON: 0.73 ± 0.19 (2^−Δct^ vs undigested gDNA), *r* = 0.08, *p* = 0.43), Fig. 4I).

**Fig. 5:**
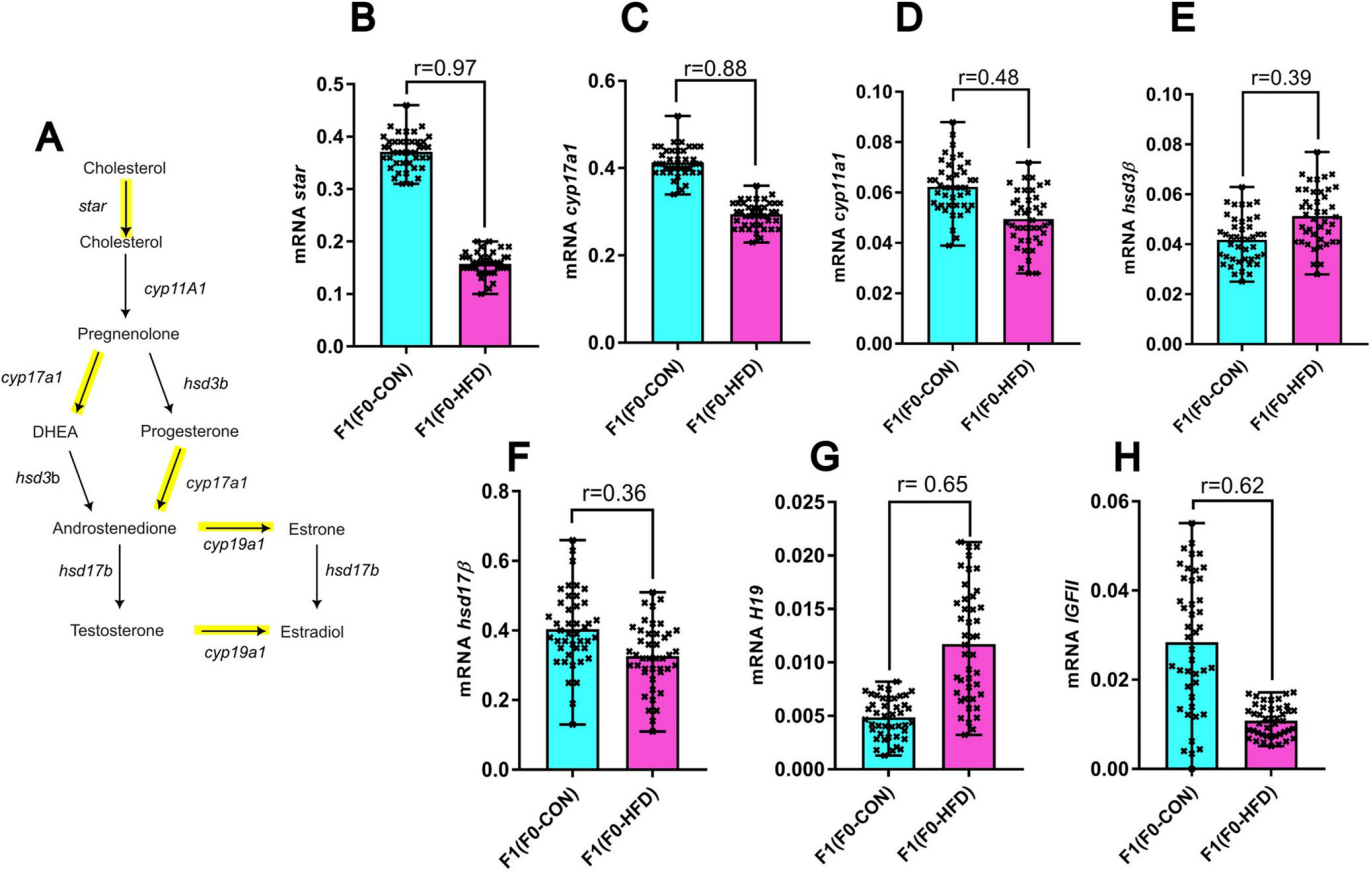
F0-HFD sires influence genes involved in testosterone homoeostasis in offspring testis. **A** Representative scheme of testosterone biosynthesis. **B**–**H** Quantitation of *star*(B), *cyp17a1*(C), *cyp11a1*(D), *hsd3β* (E), *hsd17β* (F), *H19* (**G**) and *IGFII* (**H**) transcripts in testis of F1(F0-HFD) and F1(F0-CON). For analysis in **B**–**H** fold change is expressed as 2^−^^∆ct^ vs housekeeping gene *β-actin*. For each analysis comparisons were made using a *t* test (*n* = 43, each from a different mother). Individual data are scattered as dark crosses together with their mean and range. Bars are labelled with effect size: *r* > 0.85 = large, 0.5 < *r* < 0.85 = medium, 0.2 < *r* < 0.5 = small, *r* < 0.2 = no effect.

Finally, to understand if the effect of F0-HFD could affect overall testosterone homoeostasis in testis of F1(F0-HFD), we tested the expression levels of five genes involved in the testosterone genesis (Fig. 5): steroidogenic acute regulatory protein *star* (involved in cholesterol transport), *cyp11a1* and *cyp17a1*, (two cytochromes responsible for the conversion of cholesterol into pregnenolone and of the latter in dehydroepiandrosterone, respectively), *hsd3β*, (responsible for the conversion of pregnenolone into progesterone) and *hsd17β (*responsible for the conversion of androstenedione in testosterone) (Fig. 5A). Among the tested mRNAs and compared to F1(F0-CON), F1(F0-HFD) testis present decreased transcription of *star* (F1(F0-HFD): 0.16 ± 0.02; F1(F0-CON): 0.37 ± 0.03 (2^−^^Δct^ vs β-*actin*), *r* = 0.97, *p* < 0.001) (Fig. 5B) and of *cyp17a1* (F1(F0-HFD): 0.30 ± 0.03; F1(F0-CON): 0.41 ± 0.03 (2^−^^Δct^ vs β-*actin*), *r* = 0.88, *p* < 0.001) (Fig. 5C), and less prominently modulation of the other three genes tested, suggesting paternal HFD affecting the expression of more than one gene involved in testosterone homoeostasis.

Interestingly, the change in expression of H19 and IGFII measured in offspring pancreas and induced by paternal HFD, is different from that measured for the same genes in the testis of F1(F0-HFD), suggesting a tissue specific outcome for paternal epigenetic signalling. As shown in Fig. 5 panels G, H, *H19* transcript is induced in F1(F0-HFD) testis (F1(F0-HFD): 0.0120 ± 0.005; F1(F0-CON): 0.0049 ± 0.0018 (2^−Δct^ vs β-*actin*), *r* = 0.62, *p* < 0.001) at the detriment of *IGFII* whose expression is instead reduced in F1(F0-HFD) testis (F1(F0-HFD): 0.011 ± 0.004; F1(F0-CON): 0.028 ± 0.015 (2^−Δct^ vs β-*actin*), *r* = 0.65, *p* < 0.001).

## Discussion

Before and during conception, environmental stimuli experienced by parents can be “perceived” by their germ cells, epigenetically recorded by sperms and oocytes and finally “printed” in the genome of their offspring. Parental nutrition, psychological stress, drug use/abuse, and the presence of environmental pollutants are all factors epigenetically affecting offspring genome. Probably, the evolutionary convenience of this intergenerational inheritance consists in parents predisposing their offspring to the environment these will most likely experience, allowing them to promptly pre-adapt to it^19^. This paradigm of non-Mendelian genetics, proven in rodents and humans, seems thus to swim against the genetic diversity achieved by sexual reproduction and, differently from this latter, reduces (instead of increasing) the number of biological environments the progeny will be able to adapt to. Indeed, the advantage achieved by intergenerational inheritance will be effective only when the environment experienced by the offspring will be identical to that of their parents. According to the hypothesis of Gluckman and Hanson^36^, when a mismatch between the developmental and post-developmental environments occurs, the attempt to achieve a better biological fitting can instead turn into a predisposition to dysmetabolism and dysfunctional behaviours^19^. Many authors have hypothesised that intergenerational inheritance, when fully confirmed in humans, could represent one of the undercover reasons for the modern spreading of metabolic diseases, including type II diabetes^43,44^.

While the contribution of epigenetic inheritance to the dysmetabolism of future generations is strengthen by new scientific evidence, it seems unlikely that science might find a way to avoid the occurrence of these improper epigenetic signatures. The few available clinical evidence reveal that, in humans, epigenetic signals are recorded in a broad window of time and might label spermatogonia and oogonia since puberty, and thus years before mating and conception of the progeny^45^. Even upon conception, the environment will affect developing embryos mostly in the first weeks of pregnancy, a phase in which most women are unaware of their physiological state^17^. At light of this evidence, it seems more likely that, to interrupt deleterious epigenetic intergenerational signalling, society must promote campaigns of “intergenerational prevention” and alert young adults of the intergenerational risk of unhealthy eating habits, lifestyles, and environmental pollution.

Notwithstanding, identifying genes that are repositories and/or transducers of epigenetic parental inheritance it is of pivotal interest to develop epigenetic screening able to promptly identify newborns susceptible to developing dysmetabolism in adulthood, alert them of their increased risks, and suggest preventive lifestyles, and, eventually, pharmacological treatments.

Here, we include the gene *cyp19a1* among the genes acting as epigenetic transducers of paternal intergenerational inheritance. Interestingly, murine *cyp19a1* does not contain canonical CpG islands and, thus, it has probably not been identified among other positive hits during whole-genome methylation analysis, which often focuses on CpG rich regions of the murine genome. However, the influence of methylation on *cyp19a1* has been proven in pigs^46^ fishes^47^ and humans, where differential expression of aromatase has been linked to the methylation pattern of its loci^48,49^. We show that HFD fed sires influence the methylation pattern and the preferential expression of a specific isoforms of *cyp19a1* hnRNAs, the one including the untranslated brain exon I. Interestingly, this promoter region contains a binding site for Oestrogen Receptor and for the transcription factors Jun and Fos. We prove this epigenetic alteration affect the activity of the enzyme in offspring testis, promoting hypoandrogenism and causing growth retardation and diabetes in male pups in a sexually dimorphic way. Sanchez-Garrido et al. have already shown that paternal HFD exacerbates the magnitude of HFD-induced hypogonadism in F1 males, affecting the expression of *star, hsp11 and hsd-17β*^50^, despite the authors did not investigate the activity of *cyp19a1* nor the methylation pattern of its genetic locus.

The effect exerted by the environment on *cyp19a1* activity has been already demonstrated in turtles and other reptiles^51,52^. By increasing turtle aromatase activity in eggs and promoting the conversion of testosterone into estradiol, the temperature of the nest affects the percentage of females in the progeny of *Carretta Caretta* turtles^52^. The environmental influences the methylation state and activity of this genomic locus, has been also proven in *Dicentrarchus labrax*^53^. In humans, a link between steroid hormones and intergeneration inheritance has been postulated, however, the few data available on the specific contribution of testis-produced oestrogens to dysmetabolism come from a patient with Klinefelter’s syndrome (a condition with high prevalence of metabolic syndrome and increased incidence of type 2 diabetes mellitus) where increased aromatase expression has been localised to Leydig cells and Sertoli cells. The increase in the number of aromatisation sites in these patients causes increased androgen to oestrogen conversion, with low bioavailable testosterone and high serum estradiol levels^54^.

Our study has several limitations. At first, we did not identify the molecules carrying the intergenerational signals delivered from HFD fed sires to their offspring. The altered *cy19a1* methylation pattern we measured in F1(F0-HFD) is absent in parental testis and thus likely appeared during embryo development as a consequence of trans-acting signals^55^. In our study, the genetic region we used as a positive control of intergeneration inheritance (the enhancer region in the *IGFII/H19* locus) shows a different relative expression pattern in testis and in pancreas of adult F1(F0-HFD) males. Rather than being imprinted by paternal sperm, this points toward methylation signatures appearing during embryo development or, at least, being reshuffled in a tissue specific manner^55^. We also did not investigate on the causative agent of intergenerational inheritance. F0-HFD sires present normal testosterone levels and unaltered testis morphology. Despite their mild phenotype, their *caput* epididymis sperm present increased oxidative stress and ROS levels, an environmental stimulus that has been involved in epigenetic inheritance and a likely vehicle of intergenerational transmission^56^.

A second limitation of our study is that we described the methylation pattern of *cyp19a1* only in adult testis. As previously discussed, the epigenome changes during embryo development and, in adults, it is tissue and cell specific. Further investigation will be required to understand at which stage of embryo development the *cyp19a1* methylation pattern is occurring and if it affects tissues other than testis. Even in this organ, heterogeneous in terms of cell population, we are at the moment unable to reveal in which groups of testis cells (Leydig, Sertoli, germ cells) and in which cell percentage the epigenetic change is manifesting. However, Leydig cells are responsible for the production of oestrogen and although they are the main site of aromatisation in sexually mature animals, aromatase expression and activity have also been found in germ cells, including spermatozoa^57^. It cannot therefore be excluded that germ cells are also recipient of the epigenetic information, with Leydig cells being the most influenced by the epigenetic signature^58^.

We show that paternal epigenetic signalling affects both *IGFII* and *cyp19a1*. We cannot speculate on the connection between these genes nor exclude, at the moment, the existence of a mutual relation between them in the pancreas or in the testis. Both proteins have been shown to influence pancreatic β-cell morphology and insulin secretion. IGF-II is an important gene for pancreatic β-cell function, and dictates pancreas dimension in developing embryos^59^. Intracrine testosterone activation in human pancreatic β-cells stimulates insulin secretion^60^. Both KO mice for IGF-II as well as aromatase overexpressing AROM+ mice are smaller than their wild-type littermates^58,61,62^. IGF-II in β-cells promotes type 2 diabetes^63^ as well as increases steroidogenesis in prostate cancer cells^64^. Our results seem to point toward a deleterious cumulative effect on male physiology occurring when *cyp19a1* and *IGFII/H19* epigenetic alterations are present in combination.

Despite these uncertainties, the identification of *cyp19a1* as an epigenetically controlled genomic locus might give new insights to the study of the plethora of embryonal factors increasing the risk of dysmetabolism. Indeed, when confirmed in humans, testosterone levels and *cyp19a1* activity can certainly be measured in vivo and when altered, modulated by one of the several available pharmacological inhibitors. These treatments will hardly be able to represent therapeutic strategy during the conception period. Nonetheless, during puberty, counteracting low testosterone levels due to increased aromatase activity could allow timely minimisation of hypoandrogenism, avoiding its consequence (including the onset of dysmetabolic diseases in adulthood)^65^.

Till now, most of the data on paternal intergenerational transmission of metabolic traits has been recorded using experimental platforms where HFD feeding lasted 12–20 weeks and sires presented severe dysmetabolic phenotypes. To the best of our knowledge, our data represents the first report in which a paternal mild pre-diabetic condition (occurring upon 8 weeks of HFD feeding) is sufficient to generate intergenerational transmission of dysmetabolic phenotype. If confirmed in humans, our data opens a new scenario for epidemiological research. Indeed, meta-analysis and correlation studies are so far trying to confirm paternal intergenerational inheritance in humans by measuring the rate of dysmetabolism in the progeny of fathers with manifested diabetes^45^. Our results, would, instead, point toward extending the correlation studies also to the progeny of fathers with a mild diabetic phenotype, a much larger sample size, that would very likely change the overall statistic of those studies.

## Supplementary information

Supplementary Figures

## Data Availability

All data supporting the findings of this study are available within the paper and its Supplementary Information.
