# Publisher Correction: Pre-conceptional paternal diet impacts on offspring testosterone homoeostasis via epigenetic modulation of cyp19a1/aromatase activity

**DOI:** 10.1038/s44324-025-00050-9

**Published:** 2025-03-07

**Authors:** Arianna Pastore, Nadia Badolati, Francesco Manfrevola, Serena Sagliocchi, Valentina Laurenzi, Giorgia Musto, Veronica Porreca, Melania Murolo, Teresa Chioccarelli, Roberto Ciampaglia, Valentina Vellecco, Mariarosaria Bucci, Monica Dentice, Gilda Cobellis, Mariano Stornaiuolo

**Affiliations:** 1 Department of Pharmacy, University of Naples “Federico II”, Via Montesano 49, 80131 Naples, Italy; 2 Telethon Institute of Genetics and Medicine (TIGEM), Via Campi Flegrei, 34, 80078 Pozzuoli, Italy; 3 Department of Experimental Medicine, University della Campania “Luigi Vanvitelli”, Via Santa Maria di Costantinopoli 16, 80138 Naples, Italy; 4 Department of Clinical Medicine and Surgery, Via Sergio Pansini 5, University of Naples “Federico II”, 80131 Naples, Italy

**Keywords:** Metabolic disorders, Cell biology

Correction to: *npj Metabolic Health and Disease* 10.1038/s44324-024-00011-8, published online 17 June 2024

The Data Availability statement was missing and should have read “All data supporting the findings of this study are available within the paper and its Supplementary Information.” The original article has been updated. The publisher apologises for the inconvenience caused to our authors and readers.

