# Supplementary material for: Pre-conceptional paternal diet impacts on offspring testosterone homoeostasis via epigenetic modulation of cyp19a1/aromatase activity
Source: NPJ Metab Health Dis. 2024 Jun 17;2:8. doi: 10.1038/s44324-024-00011-8 (PMC12118756; doi:10.1038/s44324-024-00011-8)
Supplement: Supplementary file 1 — Supplementary Figures [file 44324_2024_11_MOESM1_ESM.pdf]

Supplementary Figures

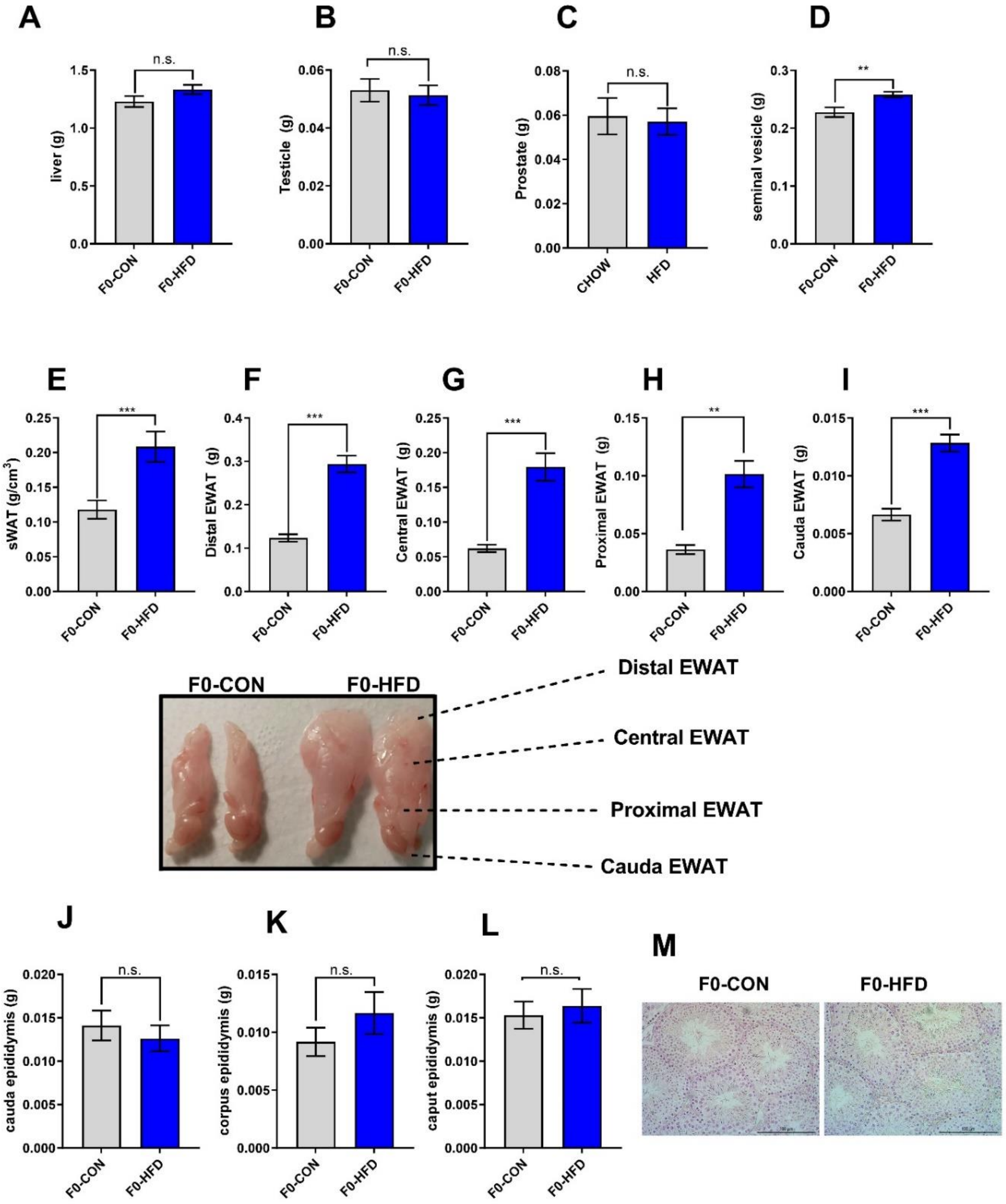

Supplementary Figure S1. **F0-HFD present increased subcutaneous and epididymal fat with minimal change in organ weight and morphology. A-D)** Compared to F0-CON, F0-HFD present increased seminal vesicles weight but not liver, testicle, and prostate weight. **E-J)** F0-HFD present increased subcutaneous (sWAT) (E) as well as epididymal (EWAT) white adipose tissue. Adiposity is significantly increased at distal (F), central (G), proximal (H) and cauda (I) epididymal eWAT sections. **J-M)** Compared to F0-CON, F0-HFD do not show significant increase in *cauda*, *corpus* and *cauda epididymis* weight nor change in overall testicle morphology as shown by the testis sections in M (scale bar = 100µm). For each analysis comparisons were made using a t test (n=30). Error bars represent SEM. Data points and bars labeled with asterisks are different at p value * < 0.05, ** < 0.01 and*** <0.001, respectively. (n.s.= non statistically significant difference).

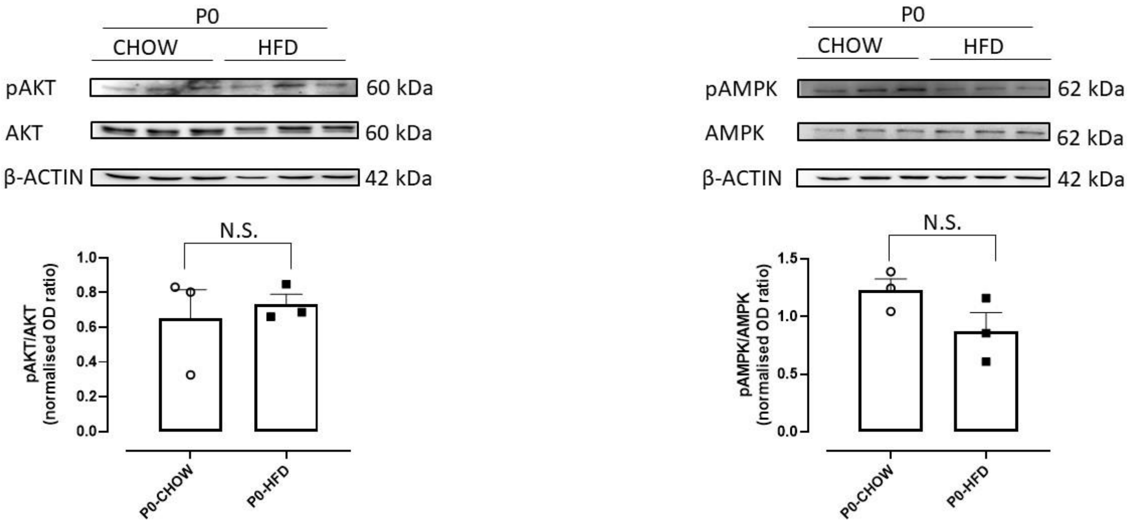

Supplementary Figure S2. **Analysis of AKT and AMPK phosphorylation confirm a mild pre-diabetic phenotype for F0-HFD animals.**

Intracellular levels of phosphorylated AKT (pAKT) and AMPK (pAMPK) measured by Western Blot in homogenates of F0-HFD (n=3) and F0-CON (n=3) livers. The bars in the graphs report the degree of phosphorylation for each protein expressed as ratio of the pAKT and pAMPK relative level and the total amount of AKT and AMPK. Sample loading was normalized by immunostaining with β-actin. Error bars represent SEM. n.s.= difference non statistically significant.

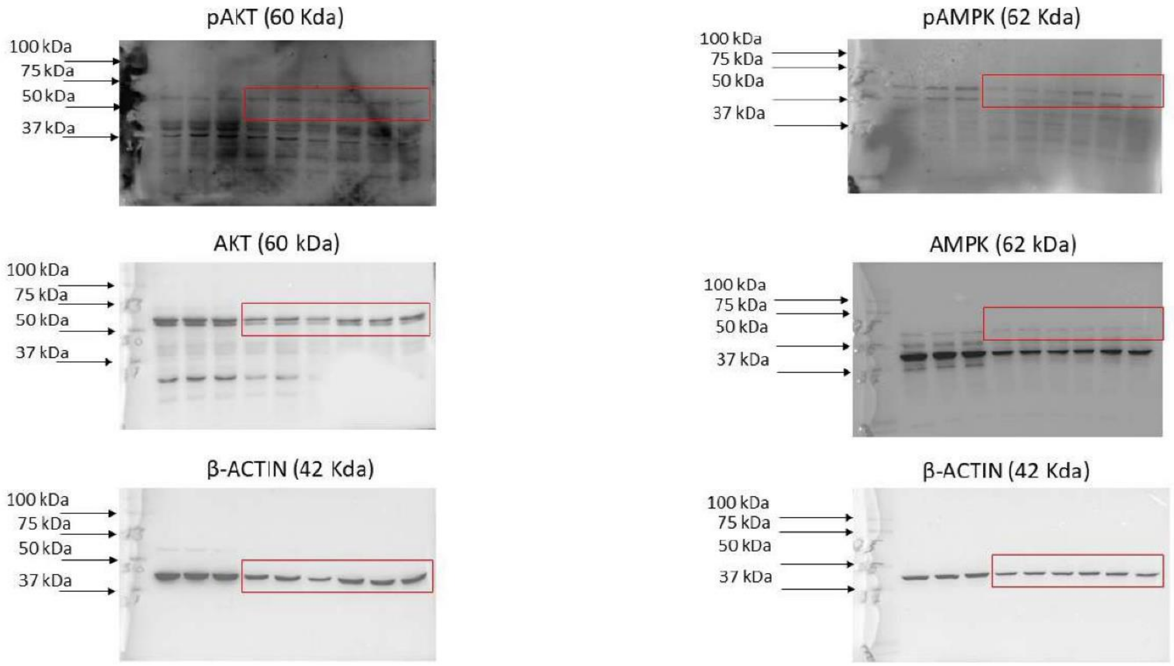

Supplementary Figure S3. **Immunoblots used for Supplementary Figure S2**

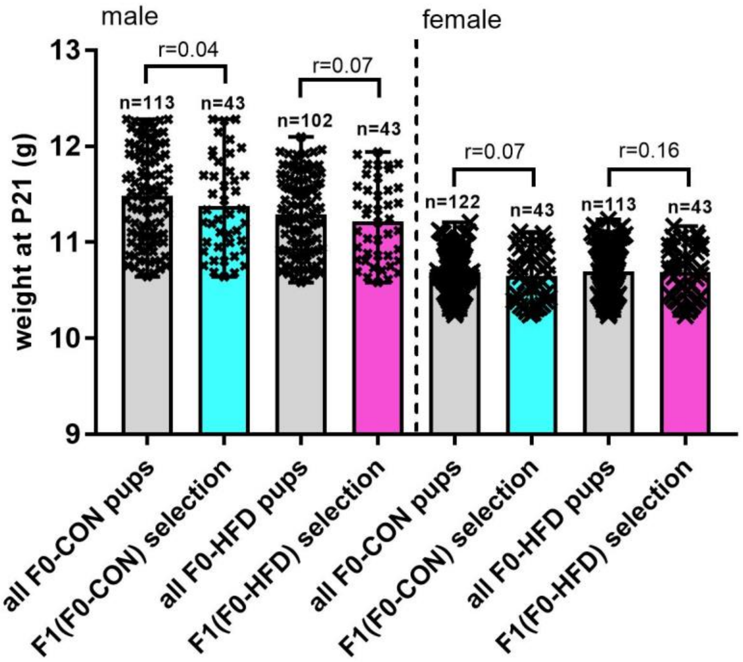

Supplementary Figure S4. Randomization of pups. Body weight at P21 of male and female pups from F0-CON or F0-HFD sires before and after selection and grouping into F1(F0-CON) and F1(F-HFD) groups. For each group n is indicated on top of the column. Individual data are presented as dark crosses together with their mean and range. Bars are labeled with effect size**:** $r > 0.85$ = large, $0.5 < r < 0.85$ = medium**,** $0.2 < r < 0.5$ = small, $r < 0.2$ = no effect.

Supplementary Table

	MATED WITH F0-CON	MATED WITH F0-HFD	effect size (r)
Female (f0 generation)	n=60	n=60	
Body weight at mating (g),	20.7 ± 1.6	20.1 ± 1.8	0.17
Diet	con	con	
Clinical biochemistry			
Fasting glucose (mg/dl)	86 ± 6	88 ± 5	0.17
Fasting insulin (pm)	103.7 ± 14.2	100.6 ± 15.1	0.10
OGTT (AUC)	36430 ± 2728	36940 ± 2820	0.09
cholesterol total (mg/dl)	105.3 ± 6.5	101.3 ± 7.1	0.28
Body weight of pups at p1 (g)	1.5 ± 0.4	1.5 ± 0.3	0
Body weight of selected pups at p1 (g)	1.5 ± 0.6	1.4 ± 0.4	0.09
Body weight of male pups at p21 (g)	11.5 ± 2.7	11.3 ± 2.2	0.04
Body weight of female pups at p21 (g)	10.7 ± 1.4	10.9 ± 1.3	0.07
Body weight of selected male pups at p21 (g) (n=43)	11.7 ± 2.2	11.6 ± 2.0	0.02
Body weight of selected female pups at p21 (g) (n=43)	10.9 ± 1.6	11.3 ± 1.1	0.14

Supplementary Table 1- Morphometric analysis, hematochemical analysis performed on adult females (n=120) mated either with F0-CON(n=60) or with F0-HFD (n=60). Results are reported as mean ± ± SD (n=60 per group). Effect size is expressed in last column as r value. Effect size : r > 0.85 = large, 0.5 < r < 0.85 = medium. 0.2 <R< 0.5 =small, r < 0.2 =no effect.

Supplementary Methods

ARRIVE guidelines 2.0

Study design	1	a) For gross morphology, hematochemical analysis, glucose tolerance we compared pairs of two groups, target group vs control group:<br>F0-CON vs F0-HFD male;<br>female mated with F0-CON vs female mated with F0-HFD;<br>F1(F0-CON) vs F1(F0-HFD) male;<br>F1(F0-CON) vs F1(F0-HFD) female.<br><br>For epigenetic and transcriptional analysis, we compared pairs of two groups, target vs control groups:<br>F0-CON vs F0-HFD male;<br>F1(F0-CON) vs F1(F0-HFD) male;<br>F1(F0-CON) vs F1(F0-HFD) female.<br><br>b) Multiple experimental units.<br><br>For all the analysis performed on F0 sires, the experimental unit is represented by a single animal. For all the analysis performed on male F1 progeny, unit is represented by a single weaned male pup. For all the analysis performed on female F1 progeny, unit is represented by a single weaned female pup. To ensure statistical independence, only one male and one female from each dam was included in the sample size.<br><br>For caloric intake measurement on F0 sires the experimental unit is represented by a cage.
Sample size	2	a) Group “male F0-CON” number of experimental unit (n) = 30<br>Group “male F0-HFD” n = 30<br>Group “female mated with F0-CON” n=60<br>Group “female mated with F0-HFD” n=60<br>Group “male F1(F0-CON)” n=113<br>Group “female F1(F0-CON)” n=165<br>Group “male F1(F0-HFD)” n=122<br>Group “female F1(F0-HFD)” n=113<br><br>Total sample size 693<br><br>Regarding the sample size in each experiment:<br>a1) for gross morphology, hematochemical analysis, glucose tolerance we compared pairs of two groups, target vs control groups: F0-CON (n=30) vs F0-HFD (n=30) male; female mated with F0-CON (n=60) vs female mated with F0-HFD (n=60)<br>To ensure statistical independence, only one male and one female from each dam was included in the sample size and thus sample size for experiment on male and female progeny was F1(F0-CON) male (n=43, non-siblings) vs F1(F0-HFD) (n=43, non-siblings) male; F1(F0-CON) (n=43, non-siblings) vs F1(F0-HFD) (n=43, non-siblings) female.<br><br>a2) for epigenetic and transcriptional analysis, we compared pairs of two groups, target vs control groups: F0-CON (n=30) vs F0-HFD (n=30) male; F1(F0-CON) male (n=43, non-siblings) vs F1(F0-HFD) male (n=43, non-siblings); F1(F0-CON) female (n=43, non-siblings) vs F1(F0-HFD) female (n=43, non-siblings).<br><br>Total sample size for the experiments 352

		b) sample size was chosen following EFSA guideline on Extended One-Generation Reproductive Toxicity Study on diet regimen Test No. 443: https://read.oecd-ilibrary.org/test-no-443
Inclusion and exclusion criteria	3	Considering the moderate risk of the procedure (high fat diet feeding) no animal were excluded during the experiments as consequence of welfare issues, ethical principles, or humane endpoints. However:<br>A1.1) At recruitment, male F0 were excluded if presenting a weight different more than 20% to the average weight of the entire sample size. Selected male F0 were randomly assigned to the F0-CON or to F0-HFD group.<br>A1.2) At recruitment, female F0 were excluded if presenting a weight different more than 20% to the average weight of the entire sample size. At recruitment, female F0 were excluded if non-nulliparous, diabetic, or hyperglycemic.<br><br>B) After births litters were reduced to 5 pups/litter when necessary to avoid underfeeding or undernutrition. No inclusion or exclusion criteria were used to select pups in overpopulated litters. Weight of selected and excluded pups are reported in the manuscript. Measurements on unselected pups are not reported.<br><br>C) n for each experiment:<br><br>For gross morphology, hematochemical analysis, glucose tolerance we compared pairs of two groups, target vs control groups: F0-CON (n=30) vs F0-HFD (n=30) male; female mated with F0-CON (n=60) vs female mated with F0-HFD (n=60)<br>To ensure statistical independence, only one male and one female from each dam was included in the sample size and thus sample size for experiment on male and female progeny was F1(F0-CON) (n=43, non-siblings) vs F1(F0-HFD) (n=43, non-siblings) male; F1(F0-CON) (n=43, non-siblings) vs F1(F0-HFD) (n=43, non-siblings) female.<br><br>For epigenetic and transcriptional analysis, we compared pairs of two groups, target vs control groups: F0-CON (n=30) vs F0-HFD (n=30) male; F1(F0-CON) (n=43, non-siblings) vs F1(F0-HFD) (n=43, non-siblings) male; F1(F0-CON) (n=43, non-siblings) vs F1(F0-HFD) (n=43, non-siblings) female.
Randomization	4	a) Allocation of F0 sires to specific groups or of F1 pups was done arbitrarily by a researcher not aware of the project. Average weight of the animals, before and after allocation or selection is reported in the manuscript as Supplementary Table 1 and Supplementary Figure S4.<br><br>b) Apart from the experimental exposure of F0 sires to a different diet (CON or HFD), the conduct of the experiment ensured no systematic difference between animals in different groups. Position of animal cages in the racks (top/middle/down) was changed twice a week to account for higher light exposure, noise or temperature fluctuation.
Blinding/masking	5	For the F0 generation. Due to the color of the diet, the experimenter could not be blinded to whether the animals were belonging to F0-CON and F0-HFD group.<br>For the animal of the F1 generation, four different investigators were involved in animal handling. The first investigator (MS) was the only person aware of the treatment group allocation. The other investigators (NB, MM, SS) were responsible for sacrifice and storage of the tissues.

		Processing and measurement were finally performed by a blinded group of investigator AP, VL, GM, FM and VP.
Outcome measured	6	a) The following parameters were assessed: gross weight; gross parameters; fasting glucose, fasting insulin, oral glucose tolerance test, AST, ALT, blood cholesterol, sperm count, sperm viability, sperm motility, sperm ROS content, mating efficiency, DNA methylation analysis, hnRNA and mRNA quantitation, gonadal hormones measurement.<br><br>b) The primary outcome of the study was to verify (and reveal the underpinning mechanistic details) dysmetabolism (assessed by gross weight, organ weight, hematochemical parameters and glucose tolerance test) in the male and female progeny of sires fed either control or hypercaloric diet. Sample size was chosen following EFSA guideline on Extended One-Generation Reproductive Toxicity Study on diet regimen Test No. 443: https://read.oecd-ilibrary.org/test-no-443
Statistical Methods	7	The study of the effect of paternal HFD on intergeneration transmission of dysmetabolism can be considered an hypothesis-testing study. However, the involvement of cyp19a1 and of its methylation status in the mechanism of intergenerational transmission is unprecedented and thus the sample size and the relative statistics must be considered exploratory and do not allow conclusions beyond the data. All the data points are reported as mean and range. Statistical analyses were performed using Prism 6 (GraphPad software). Comparisons between two groups were done by using Cohen's *d* value and the effect-size correlation.
Experimental animals	8	C57BL/6J Ola mice (n = 60), (Envigo, The Netherlands). At the start of the investigation, they were 5 weeks old and had body weights of 22.5 ± 1.1 g (F0-CON) and 22.3 ± 1.2 g (F0-HFD), respectively (mean ± SD). After 8 weeks of feeding either CON (F0-CON) or HFD (F0-HFD), sires were mated with C57BL/6J Ola female mice (n=120). At mating females had body weights of 20.7 ± 1.6 g (female mated with F0-CON) and 20.1 ± 1.8 g (female mated with F0-HFD), respectively (mean ± SD).<br>b) The C57BL/6JOla mice were supplied by Envigo (The Netherlands)
Experimental procedures	9	a-d) see Materials and Methods
Results	10	See Results
